# The developmental shift in aperiodic activity and its link to the default mode network in attention-deficit hyperactivity disorder

**DOI:** 10.1017/S0033291726104772

**Published:** 2026-06-18

**Authors:** Hui Li, Gui-Sen Wu, Yu Zhu, Chen Dang, Shao-Gen Zhong, Xi-Xi Zhao, Xiang-Sheng Luo, Yu-Feng Wang, Li Sun

**Affiliations:** 1 Peking University Sixth Hospital, Peking University Institute of Mental Health, NHC Key Laboratory of Mental Health (Peking University), National Clinical Research Center for Mental Disorders (Peking University Sixth Hospital), Beijing, China; 2 The National Clinical Research Center for Mental Disorders & Beijing Key Laboratory of Mental Disorders, Beijing Anding Hospital, Advanced Innovation Center for Human Brain Protection, Capital Medical University, Beijing, China

**Keywords:** aperiodic activity, attention-deficit/hyperactivity disorder (ADHD), default mode network (DMN), electroencephalography (EEG), neurodevelopment

## Abstract

**Background:**

Attention-deficit/hyperactivity disorder (ADHD) involves altered neurodevelopment, yet the underlying mechanisms remain elusive. Aperiodic EEG components may reflect neural functions like excitatory/inhibitory balance, but their age-related differences in ADHD and link to default mode network (DMN) dysfunction are unexplored.

**Methods:**

We included 110 medication-naïve children/adolescents with ADHD and 100 matched typically developing peers aged 6–14 years. Aperiodic parameters (exponent, offset) were derived, and source-localized alpha-band DMN coherence was computed. The sample was stratified into middle childhood (6–9 years) and early adolescence (10–14 years) subgroups to delineate age-dependent patterns.

**Results:**

ADHD showed globally increased exponent and offset versus controls. Normative age-related decreases were significantly attenuated in ADHD, indicating divergence from typical development. Age-stratified analyses revealed distinct patterns: in middle childhood, increased frontal offset correlated positively with inattention (right hemisphere) and hyperactivity/impulsivity (left hemisphere); in early adolescence, it associated with reduced coherence in two DMN pathways (right mSFG–left hippocampus and left mSFG–right MTG).

**Conclusions:**

Aperiodic activity differences in ADHD are age-dependent. Younger children exhibit focal, symptom-linked frontal abnormalities, whereas adolescents show pervasive network-level dysregulation. Aperiodic measures may capture age-varying ADHD pathophysiology, informing developmentally targeted biomarkers.

## Introduction

Attention-deficit/hyperactivity disorder (ADHD) is a prevalent neurodevelopmental condition, affecting approximately 5% of children and adolescents worldwide (Sayal et al., 2018). It is associated with significant functional impairments, including academic underachievement, social challenges, and increased risk for comorbid mental health conditions (Biederman & Faraone, 2005; Sayal et al., 2018). Despite its high prevalence and clear clinical significance, the core neurophysiological mechanisms underlying ADHD remain incompletely understood.

Electroencephalography (EEG) is particularly valuable in developmental neuroscience due to its low-cost, non-invasiveness, and high translational potential (Kappenman & Luck, 2016). EEG has been widely used to investigate ADHD, with the theta/beta ratio historically proposed as a candidate biomarker (Arns, Conners, & Kraemer, 2013). However, recent large-scale studies have failed to replicate earlier findings, demonstrating substantial group overlap and challenging its utility as a reliable marker (Boxum, Arns, & van Dijk, 2025). These limitations have prompted a methodological shift toward mathematically decomposing the EEG power spectrum, with particular focus on the aperiodic component that might capture fundamental aspects of ADHD neuropathology.

In recent years, the aperiodic component has increasingly been recognized as a stable and informative indicator of cortical microcircuit function. Analysis typically focuses on two established parameters: the exponent, which reflects the slope of spectral decay and is theorized to index the cortical excitatory/inhibitory (E/I) balance, and the offset, which represents a broadband shift in power potentially indicative of neural spiking rate (Gao, Peterson, & Voytek, 2017; Donoghue et al., 2020). Notably, these parameters evolve in a predictable manner during typical development, suggesting their utility for mapping both typical and atypical trajectories of brain maturation (Arnett et al., 2024; Hill et al., 2022; McKeon et al., 2024; Pathania et al., 2022).

Existing literature on aperiodic activity in ADHD reveals a complex and seemingly contradictory pattern, where the direction of reported abnormalities appears to depend critically on developmental stage. Young medication-naïve children exhibit steeper slopes (larger exponent) and elevated offset (Robertson et al., 2019), while adolescents tend to show flatter slopes (Karalunas et al., 2022; Ostlund, Alperin, Drew, & Karalunas, 2021). This pattern of opposing findings is further complicated by school-aged samples (ages 6–12 years), where studies have reported either a significantly lower exponent with no group differences in offset (Chen et al., 2024), or conversely, a higher exponent associated with poorer processing speed (Dakwar-Kawar et al., 2024). Recent normative data indicate that the aperiodic exponent follows a nonlinear trajectory with age (Arnett et al., 2024), suggesting that sampling across different developmental phases may partly explain the heterogeneity. However, whether aperiodic abnormalities in ADHD represent a static deficit or a dynamic deviation from typical development remains unclear.

These discrepancies may also reflect differences in sample characteristics and methodology. Many prior studies did not systematically exclude common comorbidities such as anxiety or depression (Chen et al., 2024; Karalunas et al., 2022; Ostlund, 2021; Robertson et al., 2019), included participants with prior stimulant exposure (Karalunas et al., 2022; Ostlund, 2021), or relied on whole-scalp averaging that may obscure regionally specific effects (Arnett et al., 2024; Karalunas et al., 2022; Ostlund, 2021). ADHD presentation and symptom severity are also inconsistently reported, limiting cross-study comparability.

Furthermore, a growing theoretical perspective from computational neuroscience posits that large-scale brain network dynamics may be predominantly driven by aperiodic rather than oscillatory activity, providing a conceptual basis for examining whether similar mechanisms contribute to network dysfunction in ADHD (Monchy et al., 2025). Especially, aperiodic EEG activity may share functional properties with the default mode network (DMN), as both are thought to reflect the brain’s intrinsic, ongoing neural activity during rest (Freeman, Ahlfors, & Menon, 2009; Jacob et al., 2021). Aperiodic activity, often conceptualized as a background neurophysiological signal, is thought to constitute a fundamental physiological milieu from which task-relevant rhythmic activity emerges to support cognition (Freeman, 2006; Jacob et al., 2021). Given that both aperiodic activity and the DMN are dominant during rest and reflect intrinsic brain dynamics, we hypothesized they may be physiologically coupled. This conceptual alignment suggests that aperiodic activity may serve as a local electrophysiological substrate underlying DMN functioning.

This possibility is of particular interest in ADHD, given that DMN dysfunction has been consistently implicated in the disorder (Castellanos & Aoki, 2016; Tsai, Lin, & Gau, 2024). Moreover, the DMN can be robustly characterized during eyes-closed rest, the paradigm used in the present study, which is a well-established approach for examining resting-state network dynamics (Liu et al., 2020; Patriat et al., 2013). Critically, however, this putative link has never been directly empirically tested in ADHD. The DMN therefore represents a logical and important starting point for this initial investigation. To examine this network-level association with spatial precision, we focused on source-localized alpha-band (8–13 Hz) coherence within the DMN. Alpha oscillations are the dominant rhythm during eyes-closed rest, reflect thalamocortical regulation of arousal and inhibition, and support large-scale network integration (Jensen & Bonnefond, 2026). These properties make alpha-band coherence particularly well suited for probing intrinsic DMN dynamics. Moreover, alpha activity has been extensively studied in ADHD due to its relevance to attentional processes, further supporting its selection as a candidate frequency band for examining network dysfunction in this population.

Accordingly, the present study sought to (1) characterize aperiodic parameter patterns and their associations with clinical symptoms, (2) investigate whether these parameters relate to DMN coherence, and (3) explore age-related differences in these relationships. We hypothesized that aperiodic components would reflect aberrant neurodevelopment in ADHD and that these local disturbances would be linked to impaired DMN integration, contributing to clinical symptoms.

## Methods and materials

### Participants

A total of 210 children aged 6–14 years were enrolled, including 110 medication-naïve patients with ADHD (recruited from Peking University Sixth Hospital) and 100 age- and sex-matched typically developing (TD) controls recruited from local communities. Written informed consent was obtained from all parents; written assent was collected from children ≥8 years, and verbal assent from those <8 years. This study was approved by the Medical Ethics Committee of Peking University Sixth Hospital/Institute of Mental Health under multiple approvals covering the data collection period from June 2017 to March 2024 (approval numbers: 2017-LunShen-No.20 and 2020-LunShen-No.37).

The Kiddie Schedule for Affective Disorders and Schizophrenia-Present and Lifetime Version (K-SADS-PL) based on DSM-IV criteria was used to confirm ADHD diagnosis and rule out comorbid psychiatric disorders for participants assessed before August 2022. The Chinese version of the K-SADS-PL was translated and adapted for clinical use in China. Although formal psychometric studies of the DSM-IV Chinese version have not been published, the original K-SADS-PL has demonstrated excellent reliability and validity internationally (Kaufman et al., 1997). All interviewers completed a standardized training protocol that included independent rating of 10 video-recorded K-SADS interviews under the supervision of a senior child psychiatrist. Inter-rater reliability was assessed using intraclass correlation coefficients (ICC, two-way random, absolute agreement). The acceptable threshold was ICC ≥ 0.80 for the presence/absence of ADHD diagnosis and ICC ≥ 0.75 for dimensional symptom scores. All trainees met or exceeded these thresholds. For participants assessed after August 2022, the DSM-5 version of the K-SADS-PL was used. The Chinese version of this instrument (K-SADS-PL-C DSM-5) has recently been validated in a sample of children and adolescents aged 6–18 years, demonstrating fair to excellent inter-rater reliability (0.537–1.000) and test–retest reliability (0.468–0.885) for affective and neurodevelopmental disorders (Dun et al., 2022).

ADHD symptom severity was assessed using the Chinese version of the ADHD Rating Scale-IV (ADHD-RS-IV): Home Version, completed by parents. This scale has been validated in a large sample of Chinese schoolchildren (Su et al., 2015), demonstrating satisfactory internal consistency (Cronbach’s *α* > 0.90 for both subscales), good test–retest reliability, and acceptable parent–teacher correlations. The Chinese version of the ADHD-RS-5 was under development during data collection and therefore not available for use.

Intellectual quotient was evaluated using the Wechsler Intelligence Scale for Children (WISC, Wechsler, 2003). The study protocol transitioned from the third edition (WISC-III) to the fourth edition (WISC-IV) during the data collection phase. Consequently, 19 participants with ADHD and 21 healthy controls were assessed with the WISC-III from June 2017 to October 2018, and the remainder with the WISC-IV from November 2018 through March 2024. This change is not expected to confound the results, as the WISC-III and WISC-IV are highly correlated and both provide valid estimates of full-scale IQ (Watkins, Glutting, & Lei, 2007). We further conducted sensitivity analyses controlling for WISC version as a covariate, which confirmed that the primary findings remained unchanged, indicating that the shift in IQ assessment version did not compromise the validity of the present results (see Supplementary Table S1).

All participants were medication-naïve and met the following criteria: (a) no history of head trauma with a loss of consciousness, neurological illness, or other severe disease; (b) absence of any current or lifetime comorbid psychiatric disorders (i.e., no diagnosis other than ADHD meeting DSM-IV or DSM-5 criteria, either at the time of assessment or in the past). A complete list of excluded disorders is provided in Supplementary Table S2; (c) a full-scale IQ above 80.

### EEG data acquisition and preprocessing

Resting-state EEG was recorded for 6 minutes using a 128-channel EGI HydroCel Geodesic Sensor Net, with a sampling rate of 1000 Hz and online filtering at 0.01–400 Hz, and electrode impedance was maintained below 50 kΩ. To align with our core aim of investigating the relationship between aperiodic neural activity and DMN function, the eyes-closed (EC) paradigm was selected. We acknowledge that recent studies have advocated for eyes-open or lights-off paradigms to minimize vigilance decrements in certain populations, particularly younger children who may have difficulty maintaining eye closure (Arnett, Fearey, Peisch, & Levin, 2022; Peisch & Arnett, 2024). However, EC rest provides a suitable and widely used condition for examining resting-state network dynamics. Notably, EC rest induces distinct time-varying activity patterns within the DMN, highlighting its utility for investigating intrinsic network dynamics (Liu et al., 2020). The EC condition is therefore well suited for the present study’s focus on DMN coherence. Furthermore, to assess whether drowsiness may have influenced the recordings, participants were asked immediately after the session whether they had felt sleepy during the recording. None reported significant drowsiness, and no data were excluded on this basis.

EEG data were processed using MATLAB 2021a and EEGLAB 2021.0. The preprocessing pipeline sequentially comprised: (1) downsampling to 250 Hz; (2) electrode localization based on the international 10–20 system; (3) band-pass filtering (1–40 Hz) to remove low-frequency drift and high-frequency noise; (4) identification and interpolation of bad channels: (i) signal amplitude exceeding ±150 μV for >50% of the recording duration, or (ii) correlation <0.7 with adjacent channels (within a 3-electrode radius). Bad channels were interpolated using spherical spline interpolation. On average, the ADHD group had 1.48 ± 1.29 bad channels (range: 0–5, *n* = 110) and the TD group had 1.23 ± 1.07 bad channels (range: 0–5, *n* = 100); an independent sample *t*-test confirmed no significant group difference (*t*(208) = 1.53, *p* = .128).

After channel interpolation, continuous EEG data underwent artifact rejection prior to epoch segmentation. Non-biological outliers (e.g., electrode pops) were identified as segments with signal amplitude exceeding ±100 μV or abrupt signal slope > 50 μV/ms. Transient muscle artifacts (e.g., jaw clenching, facial twitches) were detected through visual inspection of sharp, high-amplitude deflections (>80 μV). All artifact-contaminated segments were manually removed from the continuous data. Following artifact rejection, the cleaned continuous data were segmented for subsequent analyses. Any epoch containing residual artifacts exceeding ±100 μV was excluded from further analysis. After artifact rejection, the amount of clean EEG data retained (summed across epochs) was 310.05 ± 20.51 seconds in the ADHD group and 313.73 ± 18.26 seconds in the TD group. An independent sample *t*-test confirmed no significant difference between groups (*t*(208) = −1.366, *p* = .17). These results indicate comparable data quality across groups, ruling out differential data attrition as a potential confound for aperiodic parameter estimation.

Power spectral density was estimated using Welch’s averaged periodogram method (Welch, 1967) with 4-second epochs and 50% overlap. This setup yields a frequency resolution of 0.25 Hz, sufficient to distinguish major EEG oscillations (theta, alpha, beta), and provides enough cycles for stable spectral estimation. The 50% overlap is a standard procedure that increases the number of segments for averaging, which smooths the spectrum, reduces estimation variance, and improves signal-to-noise ratio. For source-level functional connectivity, we used 2-second non-overlapping epochs. This design balances frequency resolution (0.5 Hz, adequate for the alpha-band), signal stationarity (consistent with the 1–4-second quasi-stationary assumption for EEG), and sufficient cycles for reliable coherence estimation. Critically, non-overlapping epochs ensure data independence and avoid artificially inflated connectivity estimates caused by autocorrelation from overlapping windows, which would violate statistical assumptions of independent observations (Fraschini et al., 2016).

#### Source reconstruction and coherence calculation

Source analysis was conducted using a linearly constrained minimum variance beamformer (Van Veen, van Drongelen, Yuchtman, & Suzuki, 1997) implemented in Field Trip. The source model was aligned to the ICBM152 Nonlinear (MNI152) template, and head models were calculated using the boundary element method (Fuchs et al., 2002). Regions of interest were defined according to the AAL atlas (Tzourio-Mazoyer et al., 2002).

Coherence between DMN nodes—including bilateral medial superior frontal gyrus (mSFG), posterior cingulate cortex, hippocampus, and middle temporal gyrus (MTG)—was computed in the alpha-band (8–13 Hz) using magnitude-squared coherence. The selection of these four nodes was guided by the well-established tripartite architecture of the DMN (Andrews-Hanna et al., 2010) and their extensive use in previous ADHD research (Castellanos & Aoki, 2016; Uddin et al., 2008).

#### Aperiodic parameter extraction

For each source-localized signal, the power spectrum was computed via fast Fourier transform (FFT). Aperiodic parameters (exponent and offset) were extracted across 1–40 Hz using the FOOOF algorithm (Donoghue et al., 2020). Six bilateral brain regions were included: frontal (Left: FP1, F3, F7; Right: FP2, F4, F8), central (Left: C3, T3; Right: C4, T4), and posterior (Left: P3, T5, O1; Right: P4, T6, O2).

### Statistical analysis

All statistical analyses were conducted using R (version 4.5.1), with statistical significance set at *p* < .05. Demographic and clinical characteristics were compared between groups using independent sample *t*-tests for continuous variables (age, IQ, ADHD-RS scores) and Pearson’s *χ^2^* tests for categorical variables (sex). For the primary analyses of aperiodic parameters (exponent and offset analyzed separately), we used linear mixed-effects models (LMM) fitted with the lme4 (version 1.1.37) and lmerTest (version 3.1.3) packages in R. Fixed effects included group (ADHD versus TD), brain region (frontal, central, posterior), hemisphere (left, right), and all possible two-way interactions (group × region, group × hemisphere, region × hemisphere) as well as the three-way interaction (group × region × hemisphere). Age, sex, and IQ were included as covariates. Models were estimated using restricted maximum likelihood, and *p*-values for fixed effects were obtained via Satterthwaite’s approximation for denominator degrees of freedom. A crossed random-intercept structure (1 | ID) + (1 | Region:ID) was adopted based on likelihood-ratio model comparisons (Supplementary Table S3).

To examine developmental effects, we tested continuous group × age interactions and also performed exploratory age-stratified analyses (middle childhood, ≥ 6 and < 10 years, ADHD *n* = 70, TD *n* = 60; early adolescence, ≥ 10 and < 14 years, ADHD *n* = 40, TD *n* = 40). The cut-off at age 10 was chosen based on established neurodevelopmental transitions in the ADHD literature (Shaw et al., 2007), as well as epidemiological data from China showing that the highest prevalence and disability-adjusted life year rates are found in children aged 10–14 years (Jia & Han, 2025).

Partial correlations were conducted to examine associations of aperiodic parameters with age (controlling for sex and IQ), as well as associations with clinical symptoms and DMN coherence (controlling for age, sex, and IQ). For multiple comparisons, we used Bonferroni correction for post-hoc simple effects, age-parameter correlations (*α* = .0083), symptom correlations (*α* = .0125), and FDR correction (*q* < .05) for the 28 DMN coherence tests. All *p*-values are reported as raw (*p*), Bonferroni-corrected (*p_*Bonf), or FDR-corrected (*p_*FDR).

Statistical assumptions, multicollinearity diagnostics, and IQ-related sensitivity analyses confirmed the robustness of covariate adjustment. Additional sensitivity analyses (outlier removal, IQ covariate exclusion) verified the stability of primary results (Supplementary Tables S4 and S5). All model comparison details and statistical assumption tests are fully reported in Supplementary Materials.

## Results

### Demographic and clinical characteristics

Table 1 summarizes the demographic and clinical characteristics. No significant differences were observed in age or sex ratio between groups. The ADHD group had higher ADHD-RS scores and lower IQ than controls (*p* < .001).

**Table 1.** Demographic and clinical characteristics of participants with ADHD and typically developing (TD) controls Table 1. long description.

	ADHD(*n* = 110)	TD(*n* = 100)	Statistical indicators	
	M ± SD	M ± SD	*t*/ *χ* ^*2*^	p
Age(years)	9.55 ± 1.67	9.67 ± 1.78	−0.53	.60
Sex, male:female	76:34	64:36	0.61	.47
IQ	111.31 ± 11.77	120.66 ± 12.17	−5.66	<.001
ADHD Presentations, *n* (%)	Inattentive: 17 (15.45%) Combined: 93 (84.55%)			
ADHD-RS Inattention scores	20.51 ± 3.20	6.18 ± 3.23	32.26	<.001
Hyperactivity/Impulsivity scores	17.25 ± 4.79	4.03 ± 2.71	24.89	<.001
Total scores	37.76 ± 6.41	10.21 ± 5.17	34.42	<.001

*Note:* Data are presented as Mean ± Standard Deviation. ADHD-RS,ADHD Rating Scale. Independent sample *t*-tests (or Welch’s *t*-test when homogeneity of variances was violated) were used for continuous variables, and chi-square test for sex ratio. ***p < .001. The groups were well matched on age and sex, but differed significantly on IQ and all ADHD-RS symptom scores.

### Group differences in aperiodic parameters

#### Exponent

The LMM revealed a significant main effect of group (*b* = −0.080, *SE* = 0.028, *p* = .005). Group was coded with ADHD as the reference; thus, negative *b* indicates higher values in ADHD. Significant main effects of region, hemisphere, and age were also observed (all *p* < .05). No interactions involving group were significant (all *p* > .05). Model fit: marginal *R^2^* = 0.273, conditional *R^2^* = 0.777, ICC = 0.694. Full model results are provided in Supplementary Table S6, Panel A.

#### Offset

**Figure 1.**
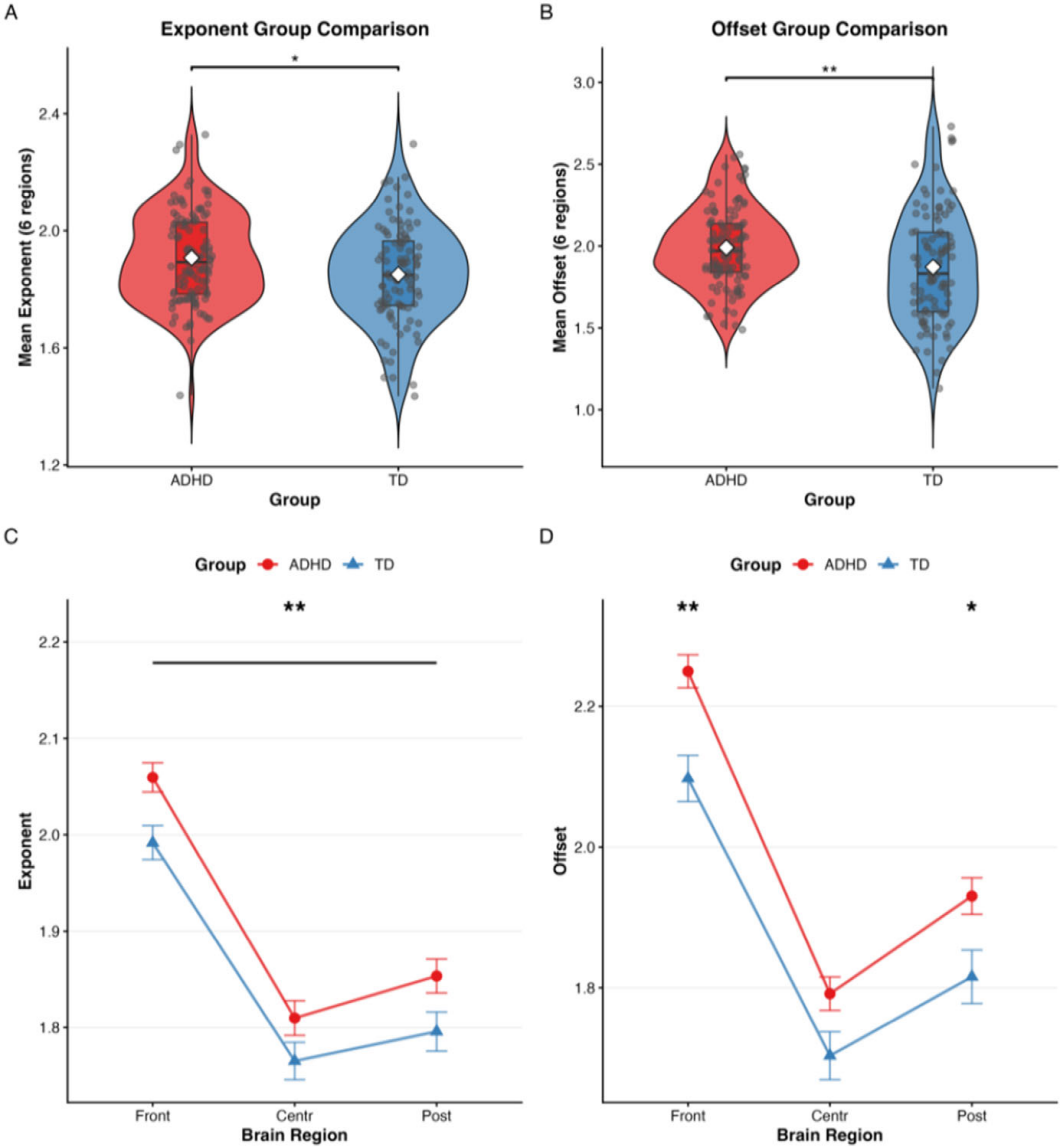
Group differences and regional patterns of aperiodic parameters. (a) Violin plots of whole-brain averaged exponent values for ADHD and TD groups. (b) Violin plots of whole-brain averaged offset values for ADHD and TD groups. (c) Line graph showing exponent values across frontal, central, and posterior regions (averaged across hemispheres). The main effect of group was significant (*p* = .005). (d) Line graph showing offset values across frontal, central, and posterior regions. Simple effect analyses with Bonferroni correction revealed a significant group difference in the frontal region (ADHD > TD, *p*_Bonf = .002) and in the posterior region (*p*_Bonf = .028). Error bars represent standard errors. ADHD = attention-deficit/hyperactivity disorder; TD = typically developing. *p < .05, **p < .01. Figure 1. long description.

A significant main effect of group was observed (*b* = −0.127, *SE* = 0.041, *p* = .002), indicating higher offset in ADHD. Main effects of region, age, and sex were also significant (all *p* < .001). With the frontal region as the reference in the model, the interaction between group and central region was significant (*b* = 0.072, *SE* = 0.028, *p* = .009), whereas the interaction between group and posterior region was not (*b* = 0.034, *SE* = 0.028, *p* = .213). Post-hoc simple effects (Bonferroni-corrected) showed a significant group difference in the frontal region (ADHD > TD, difference = 0.1245, *p*_Bonf = .002) and in the posterior region (difference = 0.0868, *p*_Bonf = .028). The central region difference was not significant. A significant region × hemisphere interaction was present (*b* = −0.048, *SE* = 0.023, *p* = .035). Model fit: marginal *R^2^* = 0.429, conditional *R^2^* = 0.897, ICC = 0.819 (see Supplementary Table S6, Panel A). These results are illustrated in Figure 1.

### Developmental effects

#### Continuous age interactions

The group × age interaction was significant for offset (*F*(1, 204) = 9.77, *p* = .002) but not significant for exponent (*p* = .063). Simple slope analysis revealed a marked age-related decrease in offset in TD group (*b* = −0.102, 95% CI [−.135, −.069], *p* < .001) that was attenuated in children with ADHD (*b* = −0.040, 95% CI [−.071, −.009], *p* = .012) (see Supplementary Figure S1 and Table S7).

#### Exploratory age-stratified analyses

**Middle childhood (6–9 years).** For exponent, a significant main effect of group was present (*b* = −0.070, *SE* = 0.035, *p* = .043). For offset, the group × central and group × posterior interactions were significant (both *p* < .05). Follow-up simple effects (Bonferroni-corrected) showed a significant group difference in the frontal region (ADHD > TD, difference = 0.098, *p*_Bonf = .039); no other region showed significant group differences. A significant main effect of sex was also observed (*p* < .001). Full model results are provided in Supplementary Table S6, Panel B.

**Figure 2.**
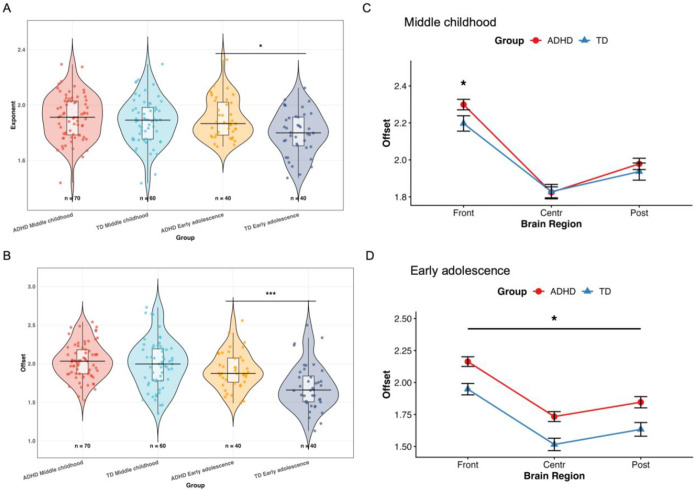
Age-stratified analyses of aperiodic parameters. (a) Violin plots of whole-brain averaged exponent values for the four subgroups: ADHD middle childhood (6–9 years), TD middle childhood, ADHD early adolescence (10–14 years), and TD early adolescence. (b) Violin plots of whole-brain averaged offset values for the same four subgroups. (c) Line graph of offset values across brain regions in the middle childhood subgroup. A significant group difference was observed in the frontal region after Bonferroni correction (ADHD > TD, *p*_Bonf = .039). (d) Line graph of offset values across brain regions in the early adolescence subgroup. The main effect of group was significant (ADHD > TD, *p* = .049). *p < .05, ***p < .001. Figure 2. long description.

**Early adolescence (10–14 years).** For exponent, the main effect of group was not significant (*p* = .072). For offset, a significant main effect of group was present (*b* = −0.142, *SE* = 0.072, *p* = .049), with significant effects of age (*p* = .037) and sex (*p* < .001). No group-related interactions were significant. A significant region × hemisphere interaction was present (*b* = −0.095, *SE* = 0.036, *p* = .010) (see Figure 2; Supplementary Table S6, Panel C).

### Correlation analysis

#### Aperiodic parameters and age

Partial correlations controlling for sex and IQ revealed significant negative associations between age and aperiodic parameters across brain regions. The TD group showed stronger negative age correlations than the ADHD group for central and posterior exponent and offset (all *p* < .05). Full results and Fisher’s *z* comparisons are provided in Supplementary Table S8.

#### Aperiodic parameters and symptoms

**Full sample.** Guided by the significant group × region interaction, we examined whether frontal offset was associated with ADHD symptoms. In the ADHD group, right frontal offset was positively correlated with inattention scores (*r*(105) = 0.262, *p*_Bonf = .024). The correlation between left frontal offset and inattention did not survive Bonferroni correction (Supplementary Table S9).

**Figure 3.**
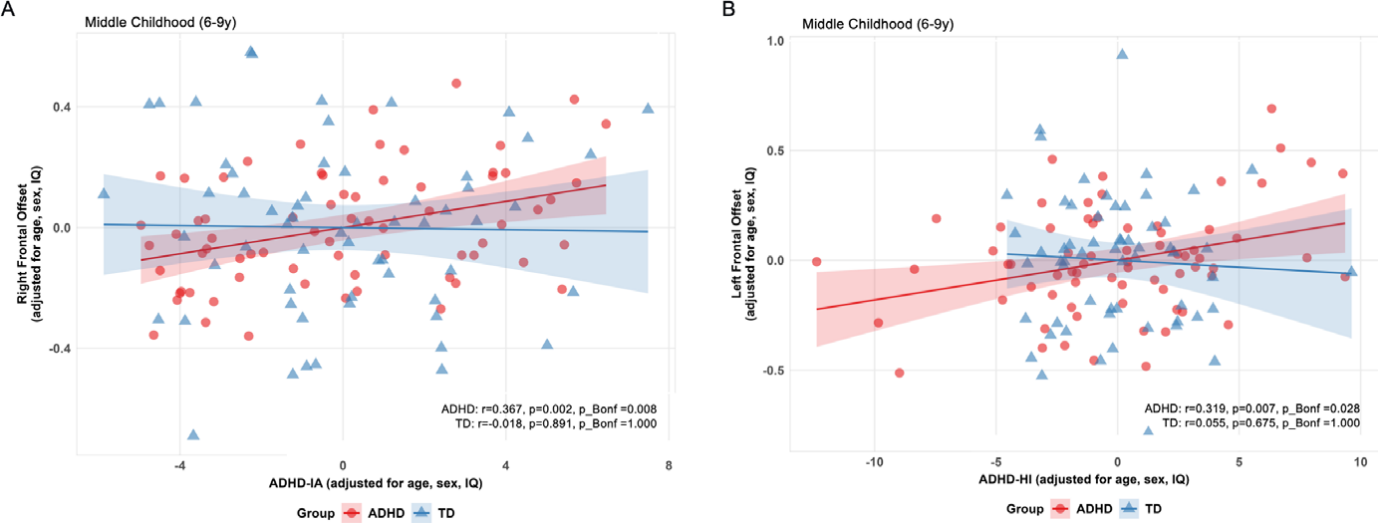
Associations between frontal offset and ADHD symptoms in middle childhood. (a) Scatter plot showing the positive correlation between right frontal offset and inattention scores in the ADHD group (*n* = 70). Partial correlation controlling for age, sex, and IQ: *r* = 0.367, *p*_Bonf = .008. (b) Scatter plot showing the positive correlation between left frontal offset and hyperactivity/impulsivity scores in the ADHD group (*n* = 70). Partial correlation: *r* = 0.319, *p*_Bonf = .028. Shaded areas represent 95% confidence intervals. No significant correlations were observed in the TD group. Figure 3. long description.

**Middle childhood**. In the ADHD group, left frontal offset was positively correlated with hyperactivity/impulsivity (*r*(65) = 0.319, *p*_Bonf = .028), and right frontal offset was positively correlated with inattention (*r*(65) = 0.367, *p*_Bonf = .008) (Figure 3). No significant correlations were observed in the TD group.

**Early adolescence**. No significant correlations survived correction in either group.

#### Aperiodic parameters and DMN coherence

**Full sample.** In the ADHD group, right frontal offset was negatively correlated with right mSFG–left hippocampus coherence (*r*(105) = −0.324, *p*_FDR = .015). No other connections were significant in either group after FDR correction (Supplementary Table S10, Panel A).

**Middle childhood**. No significant DMN correlations were found in either group.

**Figure 4.**
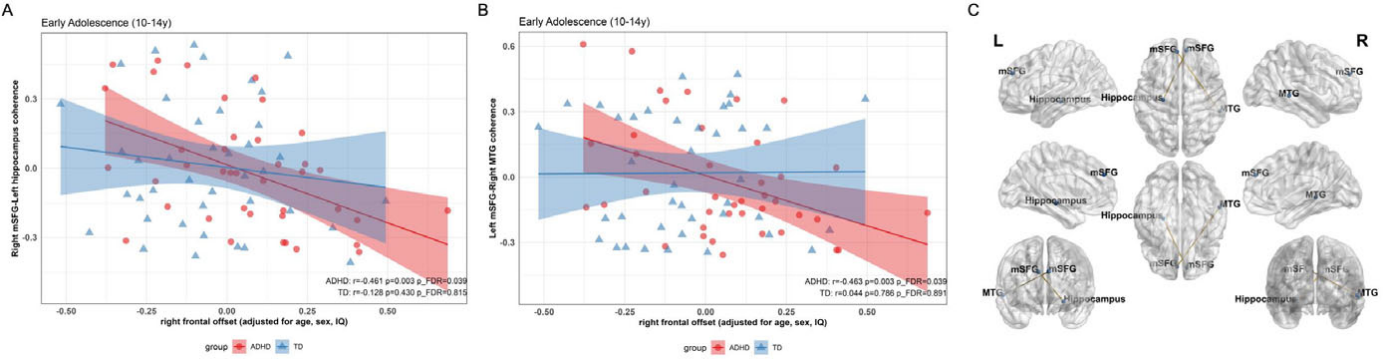
Relationship between right frontal offset and default mode network (DMN) coherence in early adolescence. (a) Scatter plot of the negative correlation between right frontal offset and alpha-band coherence of the right mSFG–left hippocampus pathway in the early adolescence ADHD group (*n* = 40). Partial correlation: *r* = −0.461, *p*_FDR = .039. (b) Scatter plot of the negative correlation between right frontal offset and alpha-band coherence of the left mSFG–right middle temporal gyrus (MTG) pathway in the same subgroup. Partial correlation: *r* = −0.463, *p*_FDR = .039. (c) Schematic illustration of the two significant DMN connections (right mSFG–left hippocampus and left mSFG–right MTG) overlaid on a standard brain template, rendered with BrainNet Viewer (Xia, Wang, & He, 2013). Figure 4. long description.

**Early adolescence.** In the ADHD group, two connections were significant: right frontal offset was negatively correlated with the right mSFG–left hippocampus coherence (*r*(35) = −0.461, *p*_FDR = .039) and the left mSFG–right MTG coherence (*r*(35) = −0.463, *p*_FDR = .039) (see Figure 4; Supplementary Table S10, Panel C).

## Discussion

The present study provides novel evidence characterizing the developmental trajectory of aperiodic EEG activity and its relationship to large-scale network function in ADHD. While the continuous interaction provides the primary statistical evidence for divergent development, age-stratified analyses illustrate that group differences appear more regionally specific in middle childhood and become more widespread by early adolescence.

At the group level, individuals with ADHD showed elevated exponent and offset, aligning with previous reports in medication-naïve young children (Robertson et al., 2019). The offset group difference was strongest in the frontal region. In middle childhood, increased frontal offset correlated with symptom severity (right with inattention, left with hyperactivity/impulsivity), consistent with the well-known role of frontal dysfunction in ADHD (Alyagon et al., 2020; Yasumura et al., 2019). In contrast, early adolescence was characterized by globally elevated aperiodic parameters without symptom correlations, potentially reflecting the emergence of more complex pathophysiological mechanisms or compensatory processes (Suskauer et al., 2008).

Our findings contrast with studies reporting reduced exponents in similar age groups (Chen et al., 2024; Karalunas et al., 2022; Ostlund, 2021). While sex composition is comparable across studies (approximately 70–75% male), several factors may contribute to these discrepancies. One key factor is comorbidity control. We excluded all current psychiatric comorbidities, whereas previous studies typically excluded only severe disorders without controlling for common conditions like anxiety and depression. Recent study found that adolescents with current depressive disorder exhibited a significantly steeper age-related decline in exponent (Woronko et al., 2026), which is opposite to the attenuated decline we observed in ADHD. Therefore, failing to control for internalizing symptoms could obscure or even reverse group differences. Another factor is medication. Our participants were completely medication-naïve, while earlier studies included many medicated individuals (27–57%). Stimulants may normalize aperiodic parameters (Karalunas et al., 2022; Robertson et al., 2019), potentially attenuating group differences in medicated samples. Besides, we used regional parcellation enabling detection of frontal-specific effects, whereas previous studies often used whole-scalp averaging, which may dilute regional effects. Similarly, other unmeasured or unreported factors, such as ADHD presentation, could contribute to cross-study variability.

Importantly, Robertson et al. (2019) reported elevated parameters in medication-naïve young children with ADHD, aligning with our middle childhood findings. Karalunas et al. (2022) proposed a developmental reversal hypothesis, whereby ADHD-related E/I balance may shift from over-inhibition in early development to over-excitation in adolescence. Our results raise the possibility that the timing of such a shift may be moderated by comorbidity or medication history, as elevated parameters persisted into early adolescence in our comorbidity-free, medication-naïve sample. These findings support a model of deviant neurodevelopment rather than simple maturational lag (Rubia, 2007; Wang et al., 2024), underscoring the importance of considering developmental stage in ADHD pathophysiology.

At the network level, this study first reveals a significant link between aperiodic EEG activity and DMN coherence in ADHD. Specifically, right frontal offset correlated negatively with alpha-band DMN coherence in an age-dependent manner. Across the full sample, higher right frontal offset predicted reduced right mSFG–left hippocampus coherence; in early adolescence, this extended to the right mSFG–left hippocampus and left mSFG–right MTG pathways. These pathways are critically involved in memory formation, contextual learning, self-referential processing, and semantic cognition (Brincat & Miller, 2015; Davey et al., 2016), all of which are frequently impaired in ADHD. Aberrant functioning within the DMN has long been implicated in inattention, mind-wandering, and poor internal control characteristic of the disorder. Our findings extend these observations by revealing that local disturbances in frontal aperiodic activity may disrupt large-scale functional integration within the DMN. Two interpretations of the age-dependent network effects are possible. The global aperiodic elevations in early adolescence could reflect worsening neural dysfunction, where local imbalances increasingly impair long-range communication. Alternatively, they could represent compensatory mechanisms, consistent with evidence that higher aperiodic parameters relate to better cognitive performance in typical adults (Montemurro et al., 2024).

### Limitations

Several limitations should be noted. First, the cross-sectional design limits inferences about within-individual developmental changes. Longitudinal and intervention studies are needed to further clarify developmental trajectories. Second, although we focused on the DMN, we do not assume these relationships are DMN-specific; future studies should extend to other networks. Third, the exclusion of comorbidities enhances internal validity but limits generalizability to the broader ADHD population. Sleep quality and socioeconomic status were not assessed and may influence results. Finally, despite source localization, EEG spatial resolution remains limited compared to fMRI.

## Conclusion

In conclusion, our multi-level analysis identifies dysregulation of fundamental cortical aperiodic activity in ADHD. While middle childhood ADHD is characterized by regionally specific frontal associations with symptoms, early adolescence ADHD exhibits more widespread group differences and emerging links to DMN coherence, providing a more nuanced pathophysiological model of ADHD. These insights carry potential clinical implications. The age-specific patterns of aperiodic dysregulation suggest that neuromodulation approaches for ADHD might benefit from developmentally informed targeting strategies. For younger children, interventions focusing on prefrontal E/I balance might be most appropriate, whereas for adolescents, approaches targeting whole-brain network integration may yield better outcomes.

## Supporting information

10.1017/S0033291726104772.sm001 Li et al. supplementary material Li et al. supplementary material

## Supplementary material

The supplementary material for this article can be found at http://doi.org/10.1017/S0033291726104772.

## Long descriptions

### Table 1. Long description

The table has five columns: variable, ADHD group with n equals 110, TD group with n equals 100, statistical indicator t or chi-squared, and p value. From top to bottom: Age in years is 9.55 plus or minus 1.67 for ADHD and 9.67 plus or minus 1.78 for TD, t equals minus 0.53, p equals 0.60. Sex male to female ratio is 76 to 34 for ADHD and 64 to 36 for TD, chi-squared equals 0.61, p equals 0.47. IQ is 111.31 plus or minus 11.77 for ADHD and 120.66 plus or minus 12.17 for TD, t equals minus 5.66, p less than 0.001. ADHD presentations for ADHD group are inattentive 17, 15.45 percent, and combined 93, 84.55 percent. No data for TD group. ADHD-RS inattention scores are 20.51 plus or minus 3.20 for ADHD and 6.18 plus or minus 3.23 for TD, t equals 32.26, p less than 0.001. Hyperactivity or impulsivity scores are 17.25 plus or minus 4.79 for ADHD and 4.03 plus or minus 2.71 for TD, t equals 24.89, p less than 0.001. Total scores are 37.76 plus or minus 6.41 for ADHD and 10.21 plus or minus 5.17 for TD, t equals 34.42, p less than 0.001. Note states data are mean plus or minus standard deviation, ADHD-RS is ADHD Rating Scale, t-tests or Welch's t-test for continuous variables, chi-squared for sex ratio, triple asterisk p less than 0.001. Groups are matched on age and sex, but differ significantly on IQ and all ADHD-RS scores.

Navigate back to Table 1..

### Figure 1. Long description

Panel A, top left, is a violin plot with y-axis labeled Mean Exponent (6 regions) from 1.2 to 2.4 and x-axis labeled Group with A D H D (red) and T D (blue). The A D H D group shows a higher mean exponent than T D, with a single asterisk above the comparison bar indicating p less than point zero five. Panel B, top right, is a violin plot with y-axis labeled Mean Offset (6 regions) from 1.0 to 3.0 and x-axis labeled Group with A D H D (red) and T D (blue). The T D group shows a higher mean offset than A D H D, with two asterisks above the comparison bar indicating p less than point zero one. Panel C, bottom left, is a line graph with y-axis labeled Exponent from 1.7 to 2.2 and x-axis labeled Brain Region with Front, Centr, and Post. Red line for A D H D and blue line for T D, both showing a decrease from Front to Centr and a slight increase at Post. The group difference is significant across regions, indicated by two asterisks above the horizontal bar. Panel D, bottom right, is a line graph with y-axis labeled Offset from 1.7 to 2.3 and x-axis labeled Brain Region with Front, Centr, and Post. Both groups decrease from Front to Centr and increase at Post, with A D H D higher than T D in Front and Post. Two asterisks above Front and one above Post indicate significant differences. Error bars represent standard errors. Legend at top of panels C and D identifies red as A D H D and blue as T D.

Navigate back to Figure 1..

### Figure 2. Long description

Panel A, top-left, is a violin plot with x-axis labeled Group and y-axis labeled Exponent. Four groups are shown: ADHD Middle childhood (n equals 70), TD Middle childhood (n equals 60), ADHD Early adolescence (n equals 40), TD Early adolescence (n equals 40). Each violin shows the distribution, median, interquartile range, and individual data points. Exponent values are similar across groups, with no significant differences marked. Panel B, bottom-left, is a violin plot with x-axis labeled Group and y-axis labeled Offset. The same four groups are shown. Offset values are higher in ADHD Early adolescence compared to TD Early adolescence, indicated by three asterisks above the violins. Panel C, top-right, is a line graph for Middle childhood, with x-axis labeled Brain Region (Front, Centr, Post) and y-axis labeled Offset. Two lines represent ADHD (red) and TD (blue). Both groups show a decrease from Front to Centr and a slight increase to Post. A significant group difference is marked by an asterisk above the Front region, with ADHD higher than TD. Panel D, bottom-right, is a line graph for Early adolescence, with the same axes and group colors. ADHD shows higher offset values than TD across all regions, with a significant main effect marked by an asterisk above the plot. Error bars indicate variability. Asterisks denote statistical significance: one asterisk for p less than point zero five, three asterisks for p less than point zero zero one.

Navigate back to Figure 2..

### Figure 3. Long description

Panel A on the left plots right frontal offset on the y axis against ADHD-I A (inattention) scores on the x axis for children aged 6 to 9 years. Red circles represent ADHD group data, blue triangles represent T D group data. The red regression line for ADHD slopes upward, indicating a positive correlation (r equals 0.367, p sub Bonf equals 0.008), with a shaded 95 percent confidence interval. The blue regression line for T D is nearly flat (r equals minus 0.018, p sub Bonf equals 1.000). Panel B on the right plots left frontal offset on the y axis against ADHD-H I (hyperactivity/impulsivity) scores on the x axis. Again, red circles are ADHD, blue triangles are T D. The red regression line for ADHD slopes upward (r equals 0.319, p sub Bonf equals 0.028), while the blue line for T D is nearly flat (r equals 0.055, p sub Bonf equals 1.000). Both panels include shaded areas for 95 percent confidence intervals. No significant correlations are observed in the T D group.

Navigate back to Figure 3..

### Figure 4. Long description

Panel A at the top-left is a scatter plot with x-axis labeled right frontal offset (adjusted for age, sex, I Q) and y-axis labeled right m S F G to left hippocampus coherence. Red circles represent A D H D group, blue triangles represent T D group. The red regression line shows a negative correlation for A D H D (r equals minus 0.461, p F D R equals 0.039), while the blue line for T D is nearly flat. Panel B at the top-right is a scatter plot with x-axis labeled right frontal offset (adjusted for age, sex, I Q) and y-axis labeled left m S F G to right M T G coherence. Red circles for A D H D and blue triangles for T D, with the red regression line showing a negative correlation for A D H D (r equals minus 0.463, p F D R equals 0.039), blue line for T D is nearly flat. Panel C on the right is a schematic brain template with labeled regions: m S F G, hippocampus, and M T G. Two pathways are highlighted: right m S F G to left hippocampus and left m S F G to right M T G, showing their spatial locations and connections.

Navigate back to Figure 4..
