# Supplementary material for: The developmental shift in aperiodic activity and its link to the default mode network in attention-deficit hyperactivity disorder
Source: Psychol Med. 2026 Jun 18;56:e199. doi: 10.1017/S0033291726104772 (PMC13280696; doi:10.1017/S0033291726104772)
Supplement: Li et al. supplementary material [file S0033291726104772sup001.docx]

**Supplementary Materials**

**1. Model Specification and Selection**

1.1 Random-Effects Structure Selection

To determine the appropriate random-effects structure, formal model comparisons were conducted. Models with by-participant random slopes for region (1 + Region | ID) failed to converge or produced singular fits, indicating overparameterization. A crossed random-intercepts structure (1 | ID) + (1 | Region:ID) was therefore tested, which provided a significantly better fit than the simple random-intercept model for both exponent (*χ²*(1) = 70.70, *p* < .001) and offset (*χ²*(1) = 46.29, *p* < .001) based on likelihood ratio tests. This structure was adopted for all primary analyses.

1.2 Model Fit Comparisons

Likelihood ratio tests confirmed that the full model (including all fixed effects) provided a significantly better fit than a null model containing only the intercept and the same random-effects structure for both exponent (*χ²*(14) = 402.37, *p* < .001) and offset (*χ²*(14) = 777.56, *p* < .001; Supplementary Table S3).

**2. IQ Covariate Diagnostics and Validation**

2.1 Rationale for IQ Inclusion

Given the significant group difference in full-scale IQ, a series of statistical diagnostics were conducted to verify the appropriateness of including IQ as a fixed-effect covariate and to address potential bias in group comparisons.

2.2 Multicollinearity Assessment

Multicollinearity was assessed via variance inflation factors (VIF) for all predictors in the primary aperiodic offset model. VIF values for group (1.74) and IQ (1.15) were well below the conservative threshold of 5, indicating negligible multicollinearity between diagnostic status and IQ.

2.3 Sensitivity Analysis for IQ Covariate

A sensitivity analysis was performed by comparing the full LMM (including age, sex, and IQ as covariates) against a reduced model excluding IQ. Key group-related effects (main effect of group and group × region interaction) were virtually identical in effect magnitude and statistical significance across models (Supplementary Table S4).

2.4 IQ Distribution Overlap

Kernel density estimation quantified the distributional overlap of IQ scores between groups. Despite a significant point-biserial correlation between diagnostic status and IQ (*r* = 0.37, *p* < .001), the IQ distributions of the ADHD and TD groups showed substantial overlap (71%; Supplementary Figure S2), confirming a shared range of IQ scores. Collectively, these diagnostics confirmed that including IQ as a fixed-effect covariate was statistically sound.

**3. Statistical Assumption Checks**

3.1 Assumptions for t-tests

For independent samples t-tests, normality was assessed using Shapiro–Wilk tests, and homogeneity of variances was examined using Levene's tests. For variables meeting homogeneity assumptions, standard t-tests were applied; when violated, Welch's t-tests were used. Although some variables deviated from normality (Shapiro–Wilk *p* < .05), t-tests are robust to such violations in large samples (*n* > 30 per group) via the central limit theorem.

3.2 Assumptions for Linear Mixed-Effects Models

For LMMs, normality of residuals was assessed via Q-Q plots and Shapiro–Wilk tests. Although significant deviations from normality were detected (all *p* < .001), visual inspection of Q-Q plots revealed only minor deviations, supporting model robustness (see Supplementary Figure S3). Homogeneity of variances was evaluated using Levene's tests on residuals, and multicollinearity was confirmed via VIF (all VIF < 2).

3.3 Assumptions for Partial Correlations

For partial correlation analyses, linearity was confirmed via scatterplot inspection. Normality of residuals was assessed using Shapiro–Wilk tests. All age-related and symptom-related partial correlations showed normally distributed residuals. For DMN coherence correlations, only right mSFG–left hippocampus coherence showed normal residuals; all others violated normality, but partial correlations are robust to moderate violations in the current sample size.

3.4 Influential Points

Influential points were examined using Cook's distance. No influential cases were detected in any analysis (all Cook's distances < 1).

**4. Sensitivity and Robustness Analyses**

Sensitivity analyses were conducted to verify the stability of primary findings. After excluding extreme outliers (standardized residuals |*z*| > 3, affecting < 1% of observations), key models were re-run. The overall pattern and statistical significance of primary results remained unchanged (Supplementary Table S5).

****Table S1.** Sensitivity Analysis: Models With and Without WISC Version**

| **Model** |  | **Predictor** | **With IQ version** | | | **Without IQ version** | | |
| --- | --- | --- | --- | --- | --- | --- | --- | --- |
|  |  |  | **Estimate** | **SE** | ***p*** | **Estimate** | **SE** | ***p*** |
| **Exponent** |  | groupTD | –0.0801 | 0.0282 | 0.005 | –0.0796 | 0.0282 | 0.005 |
|  |  | age | –0.0277 | 0.0065 | < .001 | –0.0254 | 0.0062 | < .001 |
|  |  | sexgirls | 0.0418 | 0.0228 | 0.068 | 0.0445 | 0.0227 | 0.052 |
|  |  | IQ | 0.0002 | 0.0009 | 0.798 | 0.0003 | 0.0009 | 0.751 |
|  |  | groupTD:RegionCentr | 0.0278 | 0.0262 | 0.289 | 0.0278 | 0.0262 | 0.289 |
|  |  | groupTD:RegionPost | 0.0214 | 0.0262 | 0.413 | 0.0214 | 0.0262 | 0.413 |
|  |  | groupTD:HemisphereRight | 0.0201 | 0.021 | 0.341 | 0.0201 | 0.021 | 0.341 |
|  |  | RegionPost:HemisphereRight | 0.0091 | 0.0205 | 0.656 | 0.0091 | 0.0205 | 0.656 |
| **Offset** |  | groupTD | –0.1278 | 0.041 | 0.002 | –0.1275 | 0.041 | 0.002 |
|  |  | age | –0.0731 | 0.0105 | < .001 | –0.0715 | 0.0101 | < .001 |
|  |  | sexgirls | –0.1761 | 0.0368 | < .001 | –0.1742 | 0.0366 | < .001 |
|  |  | IQ | –0.0011 | 0.0015 | 0.431 | –0.0011 | 0.0014 | 0.446 |
|  |  | groupTD:RegionCentr | 0.0721 | 0.0276 | 0.009 | 0.0721 | 0.0276 | 0.009 |
|  |  | groupTD:RegionPost | 0.0344 | 0.0276 | 0.213 | 0.0344 | 0.0276 | 0.213 |
|  |  | groupTD:HemisphereRight | 0.0061 | 0.0232 | 0.795 | 0.0061 | 0.0232 | 0.795 |
|  |  | RegionPost:HemisphereRight | –0.0479 | 0.0227 | 0.035 | –0.0479 | 0.0227 | 0.035 |

Note. This table compares key fixed‑effect estimates from linear mixed‑effects models with and without the WISC version (III vs. IV) as an additional covariate. All models already include age, sex, and IQ as covariates. Values represent fixed‑effect estimates, standard errors (SE), and *p-*values. The reference groups are ADHD (group), Left hemisphere, and Male sex. The inclusion of the WISC version did not alter the significance, direction, or magnitude of any key effects, confirming the robustness of the primary findings. TD = typically developing; Centr = Central; Post = Posterior.

| **Category** | **Conditions Excluded** |
| --- | --- |
| ****Affective Disorders**** | Major depressive disorder, dysthymia (persistent depressive disorder), bipolar I disorder, bipolar II disorder, cyclothymic disorder |
| ****Anxiety Disorders**** | Generalized anxiety disorder, separation anxiety disorder, social phobia (social anxiety disorder), specific phobia, panic disorder, agoraphobia |
| ****Obsessive-Compulsive and Related Disorders**** | Obsessive-compulsive disorder |
| ****Trauma- and Stressor-Related Disorders**** | Post-traumatic stress disorder, adjustment disorder |
| ****Disruptive, Impulse-Control, and Conduct Disorders**** | Oppositional defiant disorder, conduct disorder |
| ****Tic Disorders**** | Tourette's disorder, persistent (chronic) motor or vocal tic disorder, provisional tic disorder |
| ****Elimination Disorders**** | Enuresis (nocturnal enuresis, diurnal enuresis), encopresis |
| ****Eating Disorders**** | Anorexia nervosa, bulimia nervosa, binge-eating disorder |
| ****Psychotic Disorders**** | Schizophrenia, schizoaffective disorder, schizophreniform disorder, delusional disorder, brief psychotic disorder |
| ****Neurodevelopmental Disorders**** | Autism spectrum disorder, intellectual disability, communication disorders, motor disorders (other than tic disorders) |
| ****Medical and Neurological Conditions**** | Any history of head trauma with loss of consciousness, neurological illness (e.g., epilepsy, cerebral palsy), or other severe systemic disease that may affect brain function |

****Table S2.**** Complete List of Excluded Comorbid Disorders

*Note.* Exclusion was based on a comprehensive assessment using all modules of the Kiddie Schedule for Affective Disorders and Schizophrenia – Present and Lifetime Version (K‑SADS‑PL) for DSM‑IV (before August 2022) or DSM‑5 (after August 2022), combined with a thorough medical history interview with parents. Participants with subthreshold symptoms that did not meet full diagnostic criteria were not excluded.

****Table S3.** Model Comparison for Random-Effects Structures and Overall Fixed Effects**

| **Dependent Variable** | **Model** | **Fixed Effects** | **Random Effects** | **AIC** | **BIC** | **logLik** | **Comparison** | **χ²** | **df** | ***p*** |
| --- | --- | --- | --- | --- | --- | --- | --- | --- | --- | --- |
| Exponent | Null | Intercept only | (1 \| ID) + (1 \| Region:ID) | -805.07 | -784.51 | -409.53 | Crossed RE vs Null | 402.37 | 14 | < .001 |
|  | Simple RE | All fixed effects | (1 \| ID) | -1110.70 | -1023.40 | -572.37 | Crossed RE vs Simple RE | 70.70 | 1 | < .001 |
|  | Crossed RE | All fixed effects | (1 \| ID) + (1 \| Region:ID) | -1179.44 | -1086.94 | -607.72 | — | — | — | — |
| Offset | Null | Intercept only | (1 \| ID) + (1 \| Region:ID) | -80.50 | -59.94 | -46.25 | Crossed RE vs Null | 777.56 | 14 | < .001 |
|  | Simple RE | All fixed effects | (1 \| ID) | -785.77 | -698.41 | -409.89 | Crossed RE vs Simple RE | 46.29 | 1 | < .001 |
|  | Crossed RE | All fixed effects | (1 \| ID) + (1 \| Region:ID) | -830.06 | -737.56 | -433.03 | — | — | — | — |

*Note.* All models were estimated using REML for final reporting; likelihood ratio tests for comparisons were performed using ML estimation. AIC = Akaike Information Criterion; BIC = Bayesian Information Criterion; RE = random effects.

****Table** S4.** Sensitivity Analysis: Models With and Without IQ

| **Model** | **Predictor** | **With IQ** | | | **Without IQ** | | |
| --- | --- | --- | --- | --- | --- | --- | --- |
|  |  | **Estimate** | **SE** | ***p*** | **Estimate** | **SE** | ***p*** |
| **Exponent** | groupTD | -0.080 | 0.028 | .005 | -0.077 | 0.027 | .004 |
|  | groupTD:RegionCentr | 0.028 | 0.026 | .289 | 0.028 | 0.026 | .289 |
|  | groupTD:RegionPost | 0.021 | 0.026 | .413 | 0.021 | 0.026 | .413 |
|  | groupTD:HemisphereRight | 0.020 | 0.021 | .341 | 0.020 | 0.021 | .341 |
|  | groupTD:RegionCentr:HemisphereRight | -0.009 | 0.030 | .757 | -0.009 | 0.030 | .757 |
|  | groupTD:RegionPost:HemisphereRight | -0.023 | 0.030 | .446 | -0.023 | 0.03 | .446 |
| **Offset** | groupTD | -0.127 | 0.041 | .002 | -0.138 | 0.039 | < .001 |
|  | groupTD:RegionCentr | 0.072 | 0.028 | .009 | 0.072 | 0.028 | .009 |
|  | groupTD:RegionPost | 0.034 | 0.028 | .213 | 0.034 | 0.028 | .213 |
|  | groupTD:HemisphereRight | 0.006 | 0.023 | .795 | 0.006 | 0.023 | .795 |
|  | RegionPost:HemisphereRight | -0.048 | 0.023 | .035 | -0.048 | 0.023 | .035 |
|  | groupTD:RegionCentr:HemisphereRight | -0.016 | 0.033 | .627 | -0.016 | 0.033 | .627 |
|  | groupTD:RegionPost:HemisphereRight | 0.007 | 0.033 | .843 | 0.007 | 0.033 | .843 |

*Note.* This table compares key fixed effects from models with and without IQ as a covariate. All models included age and sex. The results show that the inclusion of IQ did not substantially alter the magnitude or significance of the primary findings.

****Table** S5.** Robustness Analysis: Outlier Removal Effects

| **Model** | **Shapiro-W** | **Shapiro-*p*** | **Max-Deviation** | **Outliers (\|z\|>3)** | **Original-F** | **Original-*p*** | **Cleaned-F** | **Cleaned-*p*** |
| --- | --- | --- | --- | --- | --- | --- | --- | --- |
| Exponent  Main | 0.985 | < .001 | 0.648 | 11 (0.87%) | 6.45 | .012 | 7.4 | .007 |
| Offset  Main | 0.994 | < .001 | 0.506 | 8 (0.63%) | 5.94 | .016 | 5.78 | .017 |

*Note.* Outliers were defined as observations with standardized residuals |*z*| > 3. Removing these extreme values (affecting <1% of the data) did not alter the significance of the group main effect, confirming the robustness of the primary findings.

****Table** S6.** Linear Mixed Model Results

## **Panel A: Full Sample (6–14 years)**

| **Predictor** | **Exponent** | | | | | | **Offset** | | | | |
| --- | --- | --- | --- | --- | --- | --- | --- | --- | --- | --- | --- |
|  | **Estimate** | **SE** | **df** | ***t*** | ***p*** | **Estimate** | | **SE** | **df** | ***t*** | ***p*** |
| (Intercept) | 2.2706 | 0.1192 | 208.693 | 19.053 | < .001 | 3.1220 | | 0.1917 | 206.596 | 16.289 | < .001 |
| groupTD | -0.0796 | 0.0282 | 436.697 | -2.822 | .005 | -0.1275 | | 0.0410 | 300.327 | -3.111 | .002 |
| RegionCentr | -0.2500 | 0.0181 | 788.355 | -13.841 | < .001 | -0.4597 | | 0.0190 | 831.516 | -24.147 | < .001 |
| RegionPost | -0.2107 | 0.0181 | 788.355 | -11.661 | < .001 | -0.2956 | | 0.0190 | 831.516 | -15.527 | < .001 |
| HemisphereRight | -0.0289 | 0.0145 | 624.000 | -1.993 | .047 | -0.0261 | | 0.0160 | 624.000 | -1.630 | .104 |
| age | -0.0254 | 0.0062 | 205.000 | -4.068 | < .001 | -0.0715 | | 0.0101 | 205.000 | -7.108 | < .001 |
| sexgirls | 0.0445 | 0.0227 | 205.000 | 1.959 | .052 | -0.1742 | | 0.0366 | 205.000 | -4.758 | < .001 |
| IQ | 0.0003 | 0.0009 | 205.000 | 0.318 | .751 | -0.0011 | | 0.0014 | 205.000 | -0.764 | .446 |
| groupTD:RegionCentr | 0.0278 | 0.0262 | 788.355 | 1.061 | .289 | 0.0721 | | 0.0276 | 831.516 | 2.615 | .009 |
| groupTD:RegionPost | 0.0214 | 0.0262 | 788.355 | 0.819 | .413 | 0.0344 | | 0.0276 | 831.516 | 1.246 | .213 |
| groupTD:HemisphereRight | 0.0201 | 0.0210 | 624.000 | 0.953 | .341 | 0.0061 | | 0.0232 | 624.000 | 0.260 | .795 |
| RegionCentr:HemisphereRight | 0.0007 | 0.0205 | 624.000 | 0.035 | .972 | 0.0029 | | 0.0227 | 624.000 | 0.128 | .898 |
| RegionPost:HemisphereRight | 0.0091 | 0.0205 | 624.000 | 0.445 | .656 | -0.0479 | | 0.0227 | 624.000 | -2.111 | .035 |
| groupTD:RegionCentr:HemisphereRight | -0.0092 | 0.0298 | 624.000 | -0.309 | .757 | -0.0160 | | 0.0329 | 624.000 | -0.486 | .627 |
| groupTD:RegionPost:HemisphereRight | -0.0227 | 0.0298 | 624.000 | -0.762 | .446 | 0.0065 | | 0.0329 | 624.000 | 0.198 | .843 |

## **Panel B: Middle Childhood (6–9 years)**

| **Predictor** | **Exponent** | | | | | **Offset** | | | | |
| --- | --- | --- | --- | --- | --- | --- | --- | --- | --- | --- |
|  | **Estimate** | **SE** | **df** | ***t*** | ***p*** | **Estimate** | **SE** | **df** | ***t*** | ***p*** |
| (Intercept) | 2.6447 | 0.1988 | 126.292 | 13.301 | < .001 | 3.3472 | 0.3187 | 125.562 | 10.503 | < .001 |
| groupTD | -0.0702 | 0.0345 | 291.898 | -2.034 | .043 | -0.1046 | 0.0493 | 193.954 | -2.122 | .035 |
| RegionCentr | -0.2620 | 0.0228 | 496.247 | -11.481 | < .001 | -0.4899 | 0.0241 | 503.340 | -20.304 | < .001 |
| RegionPost | -0.2137 | 0.0228 | 496.247 | -9.366 | < .001 | -0.3103 | 0.0241 | 503.340 | -12.858 | < .001 |
| HemisphereRight | -0.0368 | 0.0187 | 384.000 | -1.965 | .050 | -0.0593 | 0.0200 | 384.000 | -2.959 | .003 |
| age | -0.0730 | 0.0162 | 125.000 | -4.519 | < .001 | -0.1244 | 0.0259 | 125.000 | -4.799 | < .001 |
| sexgirls | 0.0594 | 0.0290 | 125.000 | 2.044 | .043 | -0.1689 | 0.0466 | 125.000 | -3.623 | < .001 |
| IQ | 0.0004 | 0.0011 | 125.000 | 0.368 | .714 | 0.0007 | 0.0018 | 125.000 | 0.414 | .679 |
| groupTD:RegionCentr | 0.0560 | 0.0336 | 496.247 | 1.666 | .096 | 0.1339 | 0.0355 | 503.340 | 3.771 | < .001 |
| groupTD:RegionPost | 0.0471 | 0.0336 | 496.247 | 1.402 | .161 | 0.0716 | 0.0355 | 503.340 | 2.017 | .044 |
| groupTD:HemisphereRight | 0.0154 | 0.0276 | 384.000 | 0.561 | .575 | 0.0138 | 0.0295 | 384.000 | 0.466 | .641 |
| RegionCentr:HemisphereRight | -0.0034 | 0.0265 | 384.000 | -0.128 | .898 | 0.0305 | 0.0283 | 384.000 | 1.077 | .282 |
| RegionPost:HemisphereRight | 0.0054 | 0.0265 | 384.000 | 0.205 | .837 | -0.0211 | 0.0283 | 384.000 | -0.746 | .456 |
| groupTD:RegionCentr:HemisphereRight | -0.0110 | 0.0390 | 384.000 | -0.284 | .777 | -0.0560 | 0.0417 | 384.000 | -1.344 | .180 |
| groupTD:RegionPost:HemisphereRight | -0.0337 | 0.0390 | 384.000 | -0.866 | .387 | -0.0224 | 0.0417 | 384.000 | -0.537 | .592 |

## **Panel C: Early Adolescence (10–14 years)**

| **Predictor** | **Exponent** | | | | | | **Offset** | | | | |
| --- | --- | --- | --- | --- | --- | --- | --- | --- | --- | --- | --- |
|  | **Estimate** | **SE** | **df** | ***t*** | ***p*** | **Estimate** | | **SE** | **df** | ***t*** | ***p*** |
| (Intercept) | 2.3894 | 0.2658 | 75.713 | 8.990 | < .001 | 3.3537 | | 0.4316 | 75.295 | 7.771 | < .001 |
| groupTD | -0.0876 | 0.0483 | 141.882 | -1.814 | .072 | -0.1421 | | 0.0716 | 101.196 | -1.984 | .049 |
| RegionCentr | -0.2291 | 0.0295 | 281.112 | -7.760 | < .001 | -0.4068 | | 0.0304 | 314.140 | -13.392 | < .001 |
| RegionPost | -0.2053 | 0.0295 | 281.112 | -6.953 | < .001 | -0.2700 | | 0.0304 | 314.140 | -8.888 | < .001 |
| HemisphereRight | -0.0153 | 0.0227 | 234.000 | -0.672 | .502 | 0.0319 | | 0.0258 | 234.000 | 1.237 | .217 |
| age | -0.0226 | 0.0160 | 75.000 | -1.419 | .160 | -0.0551 | | 0.0260 | 75.002 | -2.121 | .037 |
| sexgirls | 0.0058 | 0.0369 | 75.000 | 0.157 | .876 | -0.2246 | | 0.0600 | 75.002 | -3.746 | < .001 |
| IQ | -0.0008 | 0.0016 | 75.000 | -0.482 | .631 | -0.0045 | | 0.0026 | 75.002 | -1.766 | .082 |
| groupTD:RegionCentr | -0.0175 | 0.0417 | 281.112 | -0.419 | .675 | -0.0281 | | 0.0430 | 314.140 | -0.655 | .513 |
| groupTD:RegionPost | -0.0179 | 0.0417 | 281.112 | -0.428 | .669 | -0.0252 | | 0.0430 | 314.140 | -0.586 | .558 |
| groupTD:HemisphereRight | 0.0250 | 0.0321 | 234.000 | 0.781 | .436 | -0.0138 | | 0.0364 | 234.000 | -0.379 | .705 |
| RegionCentr:HemisphereRight | 0.0079 | 0.0321 | 234.000 | 0.246 | .806 | -0.0454 | | 0.0364 | 234.000 | -1.248 | .213 |
| RegionPost:HemisphereRight | 0.0156 | 0.0321 | 234.000 | 0.488 | .626 | -0.0947 | | 0.0364 | 234.000 | -2.600 | .010 |
| groupTD:RegionCentr:HemisphereRight | -0.0075 | 0.0454 | 234.000 | -0.164 | .870 | 0.0511 | | 0.0515 | 234.000 | 0.991 | .323 |
| groupTD:RegionPost:HemisphereRight | -0.0070 | 0.0454 | 234.000 | -0.155 | .877 | 0.0566 | | 0.0515 | 234.000 | 1.098 | .273 |

*Note.* Values represent fixed-effect estimates, standard errors (SE), degrees of freedom (df), t-statistics, and *p*-values. Reference groups: ADHD, Left hemisphere, Male sex. TD = typically developing; Centr = Central; Post = Posterior. Full model specifications and random effects are described in the Methods section.

****Table** S7**. Developmental Trajectory Models: Group × Age Interactions

| **Predictor** | **Exponent** | | | | | | **Offset** | | | | |
| --- | --- | --- | --- | --- | --- | --- | --- | --- | --- | --- | --- |
|  | **Estimate** | **SE** | **df** | ***t*** | ***p*** | **Estimate** | | **SE** | **df** | ***t*** | ***p*** |
| (Intercept) | 2.1285 | 0.1377 | 205.428 | 15.461 | < .001 | 2.7541 | | 0.2185 | 204.642 | 12.605 | < .001 |
| groupTD | 0.1637 | 0.1210 | 204.000 | 1.353 | .177 | 0.4992 | | 0.1922 | 204.000 | 2.597 | .010 |
| age | -0.0135 | 0.0088 | 204.000 | -1.531 | .127 | -0.0401 | | 0.0141 | 204.000 | -2.854 | .005 |
| RegionCentr | -0.2368 | 0.0131 | 791.877 | -18.134 | < .001 | -0.4254 | | 0.0138 | 829.497 | -30.739 | < .001 |
| RegionPost | -0.2004 | 0.0131 | 791.877 | -15.349 | < .001 | -0.2792 | | 0.0138 | 829.497 | -20.180 | < .001 |
| HemisphereRight | -0.0194 | 0.0105 | 627.000 | -1.847 | .065 | -0.0233 | | 0.0116 | 627.000 | -2.009 | .045 |
| sexgirls | 0.0447 | 0.0226 | 204.000 | 1.981 | .049 | -0.1736 | | 0.0359 | 204.000 | -4.841 | < .001 |
| IQ | 0.0005 | 0.0009 | 204.000 | 0.510 | .611 | -0.0006 | | 0.0014 | 204.000 | -0.455 | .649 |
| groupTD:age | -0.0233 | 0.0125 | 204.000 | -1.871 | .063 | -0.0618 | | 0.0198 | 204.000 | -3.125 | .002 |
| RegionCentr:HemisphereRight | -0.0037 | 0.0148 | 627.000 | -0.247 | .805 | -0.0047 | | 0.0164 | 627.000 | -0.287 | .774 |
| RegionPost:HemisphereRight | -0.0017 | 0.0148 | 627.000 | -0.112 | .911 | -0.0448 | | 0.0164 | 627.000 | -2.734 | .006 |

*Note.* This table presents results from linear mixed models testing continuous group × age interactions, controlling for region, hemisphere, age, sex, and IQ. Reference groups: ADHD, Left hemisphere, Male sex. TD = typically developing; Centr = Central; Post = Posterior. The significant interaction for offset (*p* = .002) indicates divergent developmental trajectories between ADHD and TD groups. Full model details are provided in the Methods section.

****Table** S8**. Age-Aperiodic Correlations (Full Sample) With Assumption Diagnostics

| **Region** | **Metric** | **Group** | **Partial *r*** | ***p*** | **Normality *W*** | **Normality *p*** | **Cook's Max** | **Fisher's *Z*** | ***p* (group diff)** |
| --- | --- | --- | --- | --- | --- | --- | --- | --- | --- |
| Frontal | Exponent | ADHD | -0.162 | .091 | 0.989 | .479 | 0.164 | 0.559 | .576 |
| Frontal | Exponent | TD | -0.237 | .018 | 0.986 | .383 | 0.098 |  |  |
| Frontal | Offset | ADHD | -0.287 | .002 | 0.994 | .910 | 0.096 | 1.707 | .088 |
| Frontal | Offset | TD | -0.489 | < .001 | 0.987 | .402 | 0.141 |  |  |
| Central | Exponent | ADHD | -0.076 | .43 | 0.99 | .611 | 0.176 | 2.183 | .029 |
| Central | Exponent | TD | -0.365 | < .001 | 0.983 | .226 | 0.156 |  |  |
| Central | Offset | ADHD | -0.198 | .038 | 0.975 | .035 | 0.126 | 3.155 | .002 |
| Central | Offset | TD | -0.567 | < .001 | 0.969 | .017 | 0.157 |  |  |
| Posterior | Exponent | ADHD | -0.163 | .089 | 0.988 | .412 | 0.093 | 1.933 | .053 |
| Posterior | Exponent | TD | -0.41 | < .001 | 0.99 | .630 | 0.109 |  |  |
| Posterior | Offset | ADHD | -0.297 | .002 | 0.985 | .247 | 0.141 | 2.292 | .022 |
| Posterior | Offset | TD | -0.556 | < .001 | 0.98 | .142 | 0.153 |  |  |

*Note*. Partial correlations control for sex and IQ. Normality of residuals was assessed using Shapiro‑Wilk tests; all residuals were normally distributed except for central offset in both groups (both *p* < .05), which is acceptable given the sample size. Cook's distance < 1 for all observations, indicating no influential outliers. Fisher's *z* transformation was used to compare correlation strengths between groups.

# Table S9. Partial Correlations Between Frontal Offset and ADHD Symptoms

## **Panel A: Full Sample (6–14 years)**

| **Group** | **Hemisphere** | **ADHD Scale** | **Partial *r*** | ***p*** | ***p*-Bonf** | **Normality W** | **Normality *p*** | **Cooks Max** | **Fisher's *Z*** | ***p* (diff)** |
| --- | --- | --- | --- | --- | --- | --- | --- | --- | --- | --- |
| ADHD | Left | ADHD-HI | 0.057 | .554 | 1.000 | 0.991 | .690 | 0.110 | 0.525 | .600 |
| TD | Left | ADHD-HI | -0.016 | .871 | 1.000 | 0.990 | .684 | 0.132 |  |  |
| ADHD | Left | ADHD-IA | 0.171 | .073 | .292 | 0.988 | .414 | 0.082 | 1.004 | .315 |
| TD | Left | ADHD-IA | 0.032 | .750 | 1.000 | 0.989 | .615 | 0.149 |  |  |
| ADHD | Right | ADHD-HI | 0.060 | .531 | 1.000 | 0.992 | .804 | 0.085 | 1.114 | .265 |
| TD | Right | ADHD-HI | -0.095 | .345 | 1.000 | 0.991 | .729 | 0.094 |  |  |
| ADHD | Right | ADHD-IA | 0.262 | .006 | .024 | 0.985 | .263 | 0.077 | 2.289 | .022 |
| TD | Right | ADHD-IA | -0.053 | .600 | 1.000 | 0.992 | .832 | 0.097 |  |  |

## **Panel B: Middle Childhood (6–9 years)**

| **Group** | **Hemisphere** | **ADHD Scale** | **Partial *r*** | ***p*** | ***p*-Bonf** | **Fisher's *Z*** | ***p* (diff)** |
| --- | --- | --- | --- | --- | --- | --- | --- |
| ADHD | Left | ADHD-HI | 0.319 | .007 | .028 | 2.139 | .032 |
| TD | Left | ADHD-HI | -0.055 | .675 | 1.000 |  |  |
| ADHD | Left | ADHD-IA | 0.266 | .026 | .104 | 1.705 | .088 |
| TD | Left | ADHD-IA | -0.035 | .793 | 1.000 |  |  |
| ADHD | Right | ADHD-HI | 0.243 | .042 | .168 | 2.213 | .027 |
| TD | Right | ADHD-HI | -0.149 | .255 | 1.000 |  |  |
| ADHD | Right | ADHD-IA | 0.367 | .002 | .008 | 2.239 | .025 |
| TD | Right | ADHD-IA | -0.018 | .891 | 1.000 |  |  |

## **Panel C: Early Adolescence (10–14 years)**

| **Group** | **Hemisphere** | **ADHD Scale** | **Partial *r*** | ***p*** | ***p*-Bonf** | **Fisher's *Z*** | ***p* (diff)** |
| --- | --- | --- | --- | --- | --- | --- | --- |
| ADHD | Left | ADHD-HI | -0.293 | .066 | .264 | -1.259 | .208 |
| TD | Left | ADHD-HI | -0.010 | .953 | 1.000 |  |  |
| ADHD | Left | ADHD-IA | -0.011 | .945 | 1.000 | -0.435 | .663 |
| TD | Left | ADHD-IA | 0.090 | .582 | 1.000 |  |  |
| ADHD | Right | ADHD-HI | -0.158 | .331 | 1.000 | -0.564 | .573 |
| TD | Right | ADHD-HI | -0.028 | .864 | 1.000 |  |  |
| ADHD | Right | ADHD-IA | 0.147 | .364 | 1.000 | 1.518 | .129 |
| TD | Right | ADHD-IA | -0.202 | .212 | .848 |  |  |

*Note.* Partial correlations control for age, sex, and IQ. Bonferroni correction was applied to the four correlations within the ADHD group for each cohort (corrected α = .0125). Normality of residuals was confirmed (Shapiro‑Wilk *p* > .05) and no influential outliers were detected (Cook's distance < 1). Between-group comparisons were performed using Fisher's *z* transformation.

****Table** S10.** Partial Correlations Between DMN Alpha Coherence and Right Frontal Offset

## **Panel A: Full Sample (6–14 years)**

| **Variable** | **Group** | **t** | **Normality *W*** | **Normality *p*** | **Cooks Max** | **Partial r** | **p** | **p-FDR** |
| --- | --- | --- | --- | --- | --- | --- | --- | --- |
| Right mSFG-Left hippocampus | ADHD | -3.560 | 0.979 | .086 | 0.082 | -0.324 | .001 | .015 |
| Right mSFG-Left hippocampus | TD | -2.639 | 0.977 | .072 | 0.082 | -0.258 | .010 | .271 |
| Left PCC-Right PCC | ADHD | -0.311 | 0.976 | .042 | 0.071 | -0.030 | .757 | .937 |
| Left PCC-Right PCC | TD | 0.452 | 0.958 | .003 | 0.058 | 0.046 | .652 | .954 |
| Left PCC-Left hippocampus | ADHD | -0.064 | 0.939 | <.001 | 0.060 | -0.006 | .949 | .984 |
| Left PCC-Left hippocampus | TD | 1.061 | 0.931 | <.001 | 0.097 | 0.107 | .291 | .853 |
| Left PCC-Right hippocampus | ADHD | -0.249 | 0.964 | .004 | 0.049 | -0.024 | .804 | .937 |
| Left PCC-Right hippocampus | TD | -0.840 | 0.955 | .002 | 0.046 | -0.085 | .403 | .868 |
| Left PCC-Left MTG | ADHD | -1.091 | 0.907 | <.001 | 0.107 | -0.104 | .278 | .628 |
| Left PCC-Left MTG | TD | 0.271 | 0.885 | <.001 | 0.067 | 0.027 | .787 | .954 |
| Left PCC-Right MTG | ADHD | 0.385 | 0.963 | .004 | 0.046 | 0.037 | .701 | .937 |
| Left PCC-Right MTG | TD | 0.948 | 0.955 | .002 | 0.066 | 0.095 | .346 | .868 |
| Right PCC-Left hippocampus | ADHD | 0.145 | 0.976 | .043 | 0.129 | 0.014 | .885 | .953 |
| Right PCC-Left hippocampus | TD | 0.283 | 0.974 | .045 | 0.118 | 0.029 | .778 | .954 |
| Right PCC-Right hippocampus | ADHD | -0.202 | 0.920 | <.001 | 0.063 | -0.019 | .840 | .941 |
| Right PCC-Right hippocampus | TD | -1.634 | 0.963 | .007 | 0.067 | -0.163 | .105 | .843 |
| Right PCC-Left MTG | ADHD | -2.557 | 0.954 | .001 | 0.120 | -0.239 | .012 | .152 |
| Right PCC-Left MTG | TD | 0.646 | 0.946 | <.001 | 0.091 | 0.065 | .520 | .938 |
| Right PCC-Right MTG | ADHD | -1.289 | 0.953 | .001 | 0.126 | -0.123 | .200 | .628 |
| Right PCC-Right MTG | TD | -0.103 | 0.904 | <.001 | 0.060 | -0.010 | .918 | .986 |
| Left mSFG-Left PCC | ADHD | -1.225 | 0.939 | <.001 | 0.074 | -0.117 | .223 | .628 |
| Left mSFG-Left PCC | TD | 1.122 | 0.953 | .001 | 0.090 | 0.113 | .265 | .853 |
| Left mSFG-Right PCC | ADHD | -0.018 | 0.922 | <.001 | 0.106 | -0.002 | .986 | .986 |
| Left mSFG-Right PCC | TD | 0.187 | 0.951 | .001 | 0.051 | 0.019 | .852 | .954 |
| Left mSFG-Right mSFG | ADHD | -0.372 | 0.937 | <.001 | 0.049 | -0.036 | .711 | .937 |
| Left mSFG-Right mSFG | TD | 1.468 | 0.962 | .006 | 0.053 | 0.147 | .145 | .843 |
| Left mSFG-Left hippocampus | ADHD | -1.062 | 0.961 | .003 | 0.100 | -0.102 | .290 | .628 |
| Left mSFG-Left hippocampus | TD | -0.400 | 0.941 | <.001 | 0.081 | -0.040 | .690 | .954 |
| Left mSFG-Right hippocampus | ADHD | 1.310 | 0.913 | <.001 | 0.123 | 0.125 | .193 | .628 |
| Left mSFG-Right hippocampus | TD | 0.258 | 0.914 | <.001 | 0.060 | 0.026 | .797 | .954 |
| Left mSFG-Left MTG | ADHD | -0.556 | 0.974 | .032 | 0.081 | -0.053 | .579 | .891 |
| Left mSFG-Left MTG | TD | -0.770 | 0.965 | .009 | 0.127 | -0.078 | .443 | .887 |
| Left mSFG-Right MTG | ADHD | -2.440 | 0.958 | .002 | 0.086 | -0.229 | .016 | .152 |
| Left mSFG-Right MTG | TD | -0.501 | 0.954 | .002 | 0.058 | -0.051 | .617 | .954 |
| Right mSFG-Left PCC | ADHD | -0.984 | 0.985 | .236 | 0.080 | -0.094 | .327 | .655 |
| Right mSFG-Left PCC | TD | 1.292 | 0.959 | .004 | 0.068 | 0.129 | .199 | .843 |
| Right mSFG-Right PCC | ADHD | -0.519 | 0.957 | .001 | 0.068 | -0.050 | .605 | .891 |
| Right mSFG-Right PCC | TD | -1.534 | 0.966 | .011 | 0.038 | -0.153 | .128 | .843 |
| Right mSFG-Right hippocampus | ADHD | -0.625 | 0.874 | <.001 | 0.083 | -0.060 | .533 | .878 |
| Right mSFG-Right hippocampus | TD | 0.034 | 0.881 | <.001 | 0.092 | 0.003 | .973 | .986 |
| Right mSFG-Left MTG | ADHD | -1.302 | 0.900 | <.001 | 0.091 | -0.124 | .196 | .628 |
| Right mSFG-Left MTG | TD | -1.417 | 0.942 | <.001 | 0.079 | -0.142 | .160 | .843 |
| Right mSFG-Right MTG | ADHD | -0.927 | 0.964 | .004 | 0.082 | -0.089 | .356 | .665 |
| Right mSFG-Right MTG | TD | 0.621 | 0.956 | .002 | 0.108 | 0.063 | .536 | .938 |
| Left hippocampus-Right hippocampus | ADHD | 0.699 | 0.937 | <.001 | 0.062 | 0.067 | .486 | .851 |
| Left hippocampus-Right hippocampus | TD | -0.018 | 0.952 | .001 | 0.079 | -0.002 | .986 | .986 |
| Left hippocampus-Left MTG | ADHD | -1.125 | 0.954 | <.001 | 0.071 | -0.108 | .263 | .628 |
| Left hippocampus-Left MTG | TD | -0.867 | 0.974 | .044 | 0.058 | -0.087 | .388 | .868 |
| Left hippocampus-Right MTG | ADHD | -1.494 | 0.952 | <.001 | 0.056 | -0.142 | .138 | .628 |
| Left hippocampus-Right MTG | TD | 1.260 | 0.968 | .014 | 0.080 | 0.126 | .211 | .843 |
| Right hippocampus-Left MTG | ADHD | -1.060 | 0.971 | .016 | 0.052 | -0.101 | .291 | .628 |
| Right hippocampus-Left MTG | TD | 1.032 | 0.979 | .119 | 0.087 | 0.104 | .305 | .853 |
| Right hippocampus-Right MTG | ADHD | -0.272 | 0.945 | <.001 | 0.054 | -0.026 | .786 | .937 |
| Right hippocampus-Right MTG | TD | -0.227 | 0.967 | .012 | 0.049 | -0.023 | .821 | .954 |
| Left MTG-Right MTG | ADHD | -1.181 | 0.905 | <.001 | 0.060 | -0.113 | .240 | .628 |
| Left MTG-Right MTG | TD | -0.294 | 0.939 | <.001 | 0.099 | -0.030 | .769 | .954 |

## Panel B: Middle Childhood (6–9 years)

| **Variable** | **Group** | **t** | **Partial r** | **p** | **p-FDR** |
| --- | --- | --- | --- | --- | --- |
| Right mSFG-Left hippocampus | ADHD | -1.775 | -0.210 | .080 | .750 |
| Right mSFG-Left hippocampus | TD | -2.482 | -0.310 | .016 | .448 |
| Left PCC-Right PCC | ADHD | -0.416 | -0.050 | .679 | .852 |
| Left PCC-Right PCC | TD | 1.038 | 0.135 | .304 | .774 |
| Left PCC-Left hippocampus | ADHD | -0.447 | -0.054 | .656 | .852 |
| Left PCC-Left hippocampus | TD | 1.913 | 0.244 | .061 | .539 |
| Left PCC-Right hippocampus | ADHD | -0.583 | -0.071 | .562 | .852 |
| Left PCC-Right hippocampus | TD | -0.082 | -0.011 | .935 | .979 |
| Left PCC-Left MTG | ADHD | -0.204 | -0.025 | .839 | .906 |
| Left PCC-Left MTG | TD | 0.408 | 0.054 | .685 | .896 |
| Left PCC-Right MTG | ADHD | 0.346 | 0.042 | .731 | .852 |
| Left PCC-Right MTG | TD | 1.030 | 0.134 | .307 | .774 |
| Right PCC-Left hippocampus | ADHD | 0.957 | 0.115 | .342 | .852 |
| Right PCC-Left hippocampus | TD | -0.027 | -0.004 | .979 | .979 |
| Right PCC-Right hippocampus | ADHD | 0.943 | 0.114 | .349 | .852 |
| Right PCC-Right hippocampus | TD | -0.636 | -0.083 | .528 | .869 |
| Right PCC-Left MTG | ADHD | -1.167 | -0.140 | .247 | .852 |
| Right PCC-Left MTG | TD | 0.751 | 0.098 | .456 | .851 |
| Right PCC-Right MTG | ADHD | -0.672 | -0.081 | .504 | .852 |
| Right PCC-Right MTG | TD | -0.066 | -0.009 | .948 | .979 |
| Left mSFG-Left PCC | ADHD | -0.200 | -0.024 | .842 | .906 |
| Left mSFG-Left PCC | TD | 0.665 | 0.087 | .509 | .869 |
| Left mSFG-Right PCC | ADHD | -0.472 | -0.057 | .638 | .852 |
| Left mSFG-Right PCC | TD | 0.330 | 0.043 | .743 | .896 |
| Left mSFG-Right mSFG | ADHD | 0.701 | 0.085 | .486 | .852 |
| Left mSFG-Right mSFG | TD | 1.097 | 0.143 | .277 | .774 |
| Left mSFG-Left hippocampus | ADHD | -0.011 | -0.001 | .991 | .991 |
| Left mSFG-Left hippocampus | TD | 0.296 | 0.039 | .768 | .896 |
| Left mSFG-Right hippocampus | ADHD | 2.582 | 0.299 | .012 | .168 |
| Left mSFG-Right hippocampus | TD | 0.941 | 0.123 | .351 | .774 |
| Left mSFG-Left MTG | ADHD | -0.589 | -0.071 | .558 | .852 |
| Left mSFG-Left MTG | TD | 0.322 | 0.042 | .748 | .896 |
| Left mSFG-Right MTG | ADHD | -0.365 | -0.044 | .716 | .852 |
| Left mSFG-Right MTG | TD | -0.928 | -0.121 | .357 | .774 |
| Right mSFG-Left PCC | ADHD | -0.352 | -0.043 | .726 | .852 |
| Right mSFG-Left PCC | TD | 1.200 | 0.156 | .235 | .774 |
| Right mSFG-Right PCC | ADHD | 0.489 | 0.059 | .626 | .852 |
| Right mSFG-Right PCC | TD | -1.819 | -0.232 | .074 | .539 |
| Right mSFG-Right hippocampus | ADHD | 1.212 | 0.145 | .230 | .852 |
| Right mSFG-Right hippocampus | TD | 1.005 | 0.131 | .319 | .774 |
| Right mSFG-Left MTG | ADHD | 0.405 | 0.049 | .686 | .852 |
| Right mSFG-Left MTG | TD | -1.012 | -0.132 | .316 | .774 |
| Right mSFG-Right MTG | ADHD | -0.133 | -0.016 | .894 | .927 |
| Right mSFG-Right MTG | TD | 0.413 | 0.054 | .681 | .896 |
| Left hippocampus-Right hippocampus | ADHD | 2.691 | 0.310 | .009 | .168 |
| Left hippocampus-Right hippocampus | TD | 0.549 | 0.072 | .585 | .896 |
| Left hippocampus-Left MTG | ADHD | 0.564 | 0.068 | .575 | .852 |
| Left hippocampus-Left MTG | TD | -0.453 | -0.059 | .652 | .896 |
| Left hippocampus-Right MTG | ADHD | -1.100 | -0.132 | .275 | .852 |
| Left hippocampus-Right MTG | TD | 0.920 | 0.120 | .362 | .774 |
| Right hippocampus-Left MTG | ADHD | -0.721 | -0.087 | .473 | .852 |
| Right hippocampus-Left MTG | TD | 1.800 | 0.230 | .077 | .539 |
| Right hippocampus-Right MTG | ADHD | 1.189 | 0.143 | .239 | .852 |
| Right hippocampus-Right MTG | TD | -0.872 | -0.114 | .387 | .774 |
| Left MTG-Right MTG | ADHD | 0.883 | 0.106 | .380 | .852 |
| Left MTG-Right MTG | TD | 0.077 | 0.010 | .939 | .979 |

## Panel C: Early Adolescence (10–14 years)

| **Variable** | **Group** | ***t*** | **Partial *r*** | ***p*** | ***p*-FDR** |
| --- | --- | --- | --- | --- | --- |
| Right mSFG-Left hippocampus | ADHD | -3.203 | -0.461 | .003 | .039 |
| Right mSFG-Left hippocampus | TD | -0.797 | -0.128 | .430 | .815 |
| Left PCC-Right PCC | ADHD | -0.017 | -0.003 | .987 | .987 |
| Left PCC-Right PCC | TD | -0.215 | -0.035 | .831 | .894 |
| Left PCC-Left hippocampus | ADHD | 0.670 | 0.108 | .507 | .676 |
| Left PCC-Left hippocampus | TD | -0.501 | -0.081 | .619 | .875 |
| Left PCC-Right hippocampus | ADHD | 0.316 | 0.051 | .754 | .867 |
| Left PCC-Right hippocampus | TD | -0.990 | -0.159 | .328 | .815 |
| Left PCC-Left MTG | ADHD | -1.239 | -0.197 | .223 | .481 |
| Left PCC-Left MTG | TD | 0.431 | 0.070 | .669 | .875 |
| Left PCC-Right MTG | ADHD | -0.562 | -0.091 | .577 | .735 |
| Left PCC-Right MTG | TD | 0.689 | 0.111 | .495 | .815 |
| Right PCC-Left hippocampus | ADHD | -0.019 | -0.003 | .985 | .987 |
| Right PCC-Left hippocampus | TD | 0.692 | 0.112 | .493 | .815 |
| Right PCC-Right hippocampus | ADHD | -1.273 | -0.202 | .211 | .481 |
| Right PCC-Right hippocampus | TD | -1.782 | -0.278 | .083 | .815 |
| Right PCC-Left MTG | ADHD | -2.450 | -0.369 | .019 | .177 |
| Right PCC-Left MTG | TD | 0.405 | 0.066 | .687 | .875 |
| Right PCC-Right MTG | ADHD | -1.018 | -0.163 | .315 | .536 |
| Right PCC-Right MTG | TD | -0.096 | -0.015 | .924 | .935 |
| Left mSFG-Left PCC | ADHD | -1.570 | -0.247 | .125 | .409 |
| Left mSFG-Left PCC | TD | 1.599 | 0.251 | .118 | .815 |
| Left mSFG-Right PCC | ADHD | -0.390 | -0.063 | .698 | .850 |
| Left mSFG-Right PCC | TD | 0.261 | 0.042 | .795 | .891 |
| Left mSFG-Right mSFG | ADHD | -1.707 | -0.267 | .096 | .388 |
| Left mSFG-Right mSFG | TD | 0.855 | 0.137 | .398 | .815 |
| Left mSFG-Left hippocampus | ADHD | -0.726 | -0.117 | .472 | .661 |
| Left mSFG-Left hippocampus | TD | -1.033 | -0.165 | .308 | .815 |
| Left mSFG-Right hippocampus | ADHD | -0.248 | -0.040 | .805 | .867 |
| Left mSFG-Right hippocampus | TD | -0.716 | -0.115 | .478 | .815 |
| Left mSFG-Left MTG | ADHD | 0.257 | 0.042 | .798 | .867 |
| Left mSFG-Left MTG | TD | -1.437 | -0.227 | .159 | .815 |
| Left mSFG-Right MTG | ADHD | -3.216 | -0.463 | .003 | .039 |
| Left mSFG-Right MTG | TD | 0.274 | 0.044 | .786 | .891 |
| Right mSFG-Left PCC | ADHD | -0.996 | -0.159 | .326 | .536 |
| Right mSFG-Left PCC | TD | 0.419 | 0.068 | .678 | .875 |
| Right mSFG-Right PCC | ADHD | -0.833 | -0.134 | .410 | .638 |
| Right mSFG-Right PCC | TD | 0.082 | 0.013 | .935 | .935 |
| Right mSFG-Right hippocampus | ADHD | -1.260 | -0.200 | .215 | .481 |
| Right mSFG-Right hippocampus | TD | -0.914 | -0.147 | .366 | .815 |
| Right mSFG-Left MTG | ADHD | -1.702 | -0.266 | .097 | .388 |
| Right mSFG-Left MTG | TD | -0.570 | -0.092 | .572 | .875 |
| Right mSFG-Right MTG | ADHD | -0.783 | -0.126 | .439 | .646 |
| Right mSFG-Right MTG | TD | 1.008 | 0.161 | .320 | .815 |
| Left hippocampus-Right hippocampus | ADHD | -2.146 | -0.329 | .038 | .215 |
| Left hippocampus-Right hippocampus | TD | -0.745 | -0.120 | .461 | .815 |
| Left hippocampus-Left MTG | ADHD | -1.179 | -0.188 | .246 | .491 |
| Left hippocampus-Left MTG | TD | -0.262 | -0.042 | .795 | .891 |
| Left hippocampus-Right MTG | ADHD | -1.484 | -0.234 | .146 | .409 |
| Left hippocampus-Right MTG | TD | 0.844 | 0.136 | .404 | .815 |
| Right hippocampus-Left MTG | ADHD | -1.086 | -0.174 | .284 | .530 |
| Right hippocampus-Left MTG | TD | -0.950 | -0.152 | .348 | .815 |
| Right hippocampus-Right MTG | ADHD | -1.537 | -0.242 | .133 | .409 |
| Right hippocampus-Right MTG | TD | 1.045 | 0.167 | .303 | .815 |
| Left MTG-Right MTG | ADHD | -2.167 | -0.332 | .037 | .215 |
| Left MTG-Right MTG | TD | -1.386 | -0.219 | .174 | .815 |

***Note.*** Partial correlations control for age, sex, and IQ. FDR correction was applied to the four correlations within the ADHD group for each cohort. All residuals met normality assumptions (Shapiro-Wilk *p* > .05) and no influential outliers were detected (Cook's distance < 1). Between-group comparisons were performed using Fisher's *z* transformation.


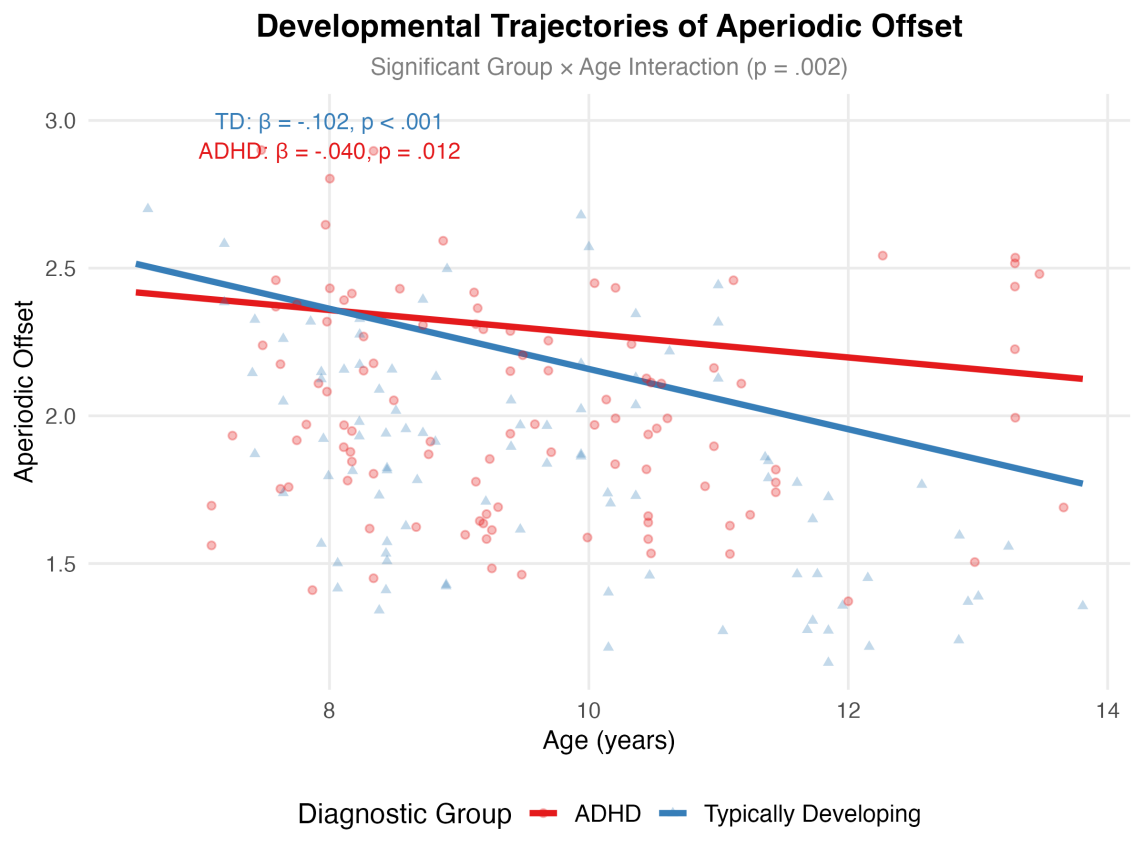


**Figure S1.** Age-related differences in aperiodic offset between the TD and ADHD groups.

Simple slope analysis revealed a significant negative association between age and offset in the TD group (*β* = -0.102, 95% CI [-0.135, -0.069], *p* < .001), which was weaker in children with ADHD (*β* = -0.040, 95% CI [-0.071, -0.009], *p* = .012).


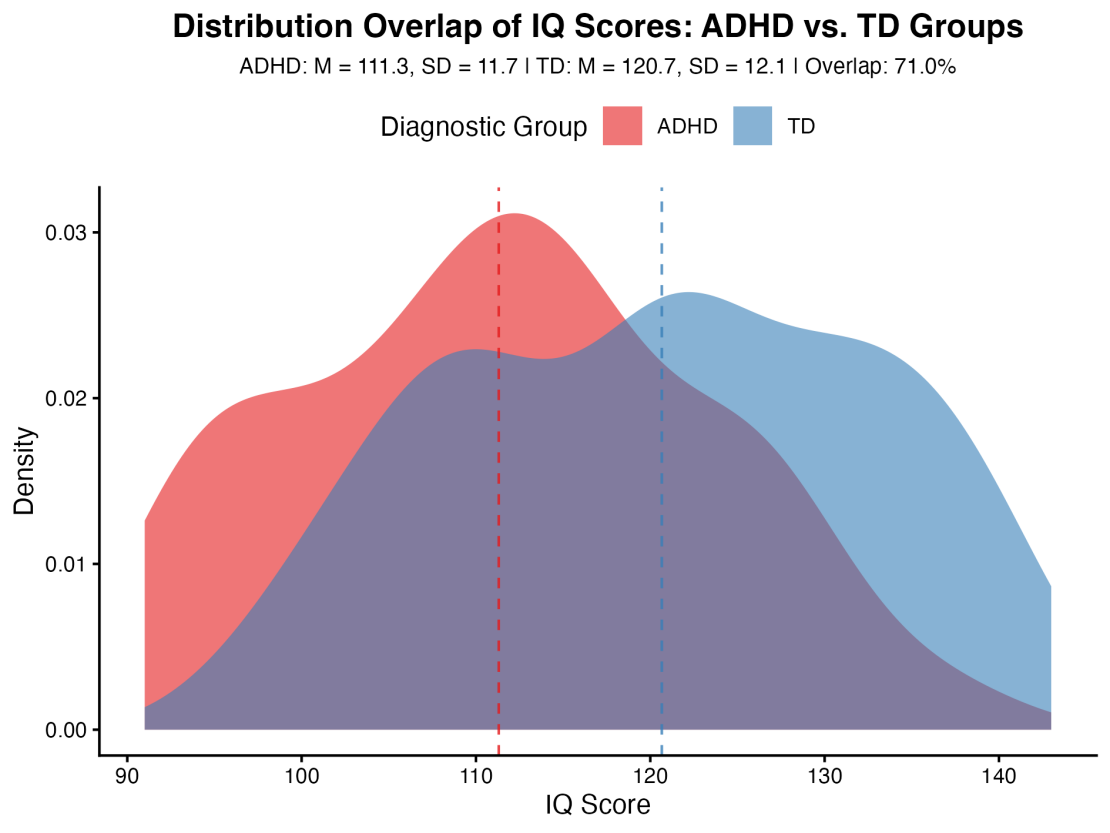
**Figure S2.** IQ Distribution Overlap Between ADHD and TD Groups. Kernel density estimation shows substantial overlap between groups, supporting the use of IQ as a covariate in the regression models despite group mean differences.

A


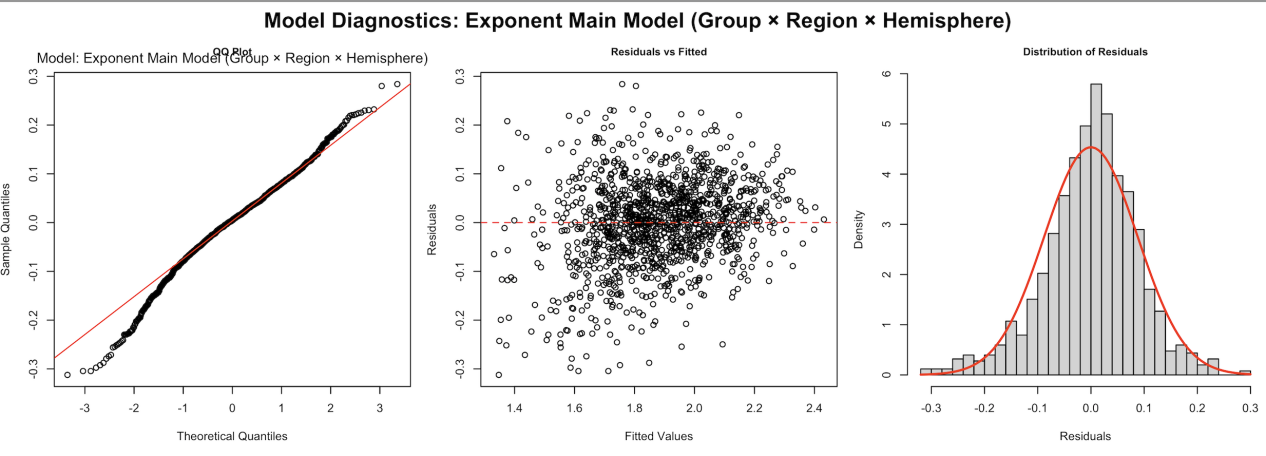

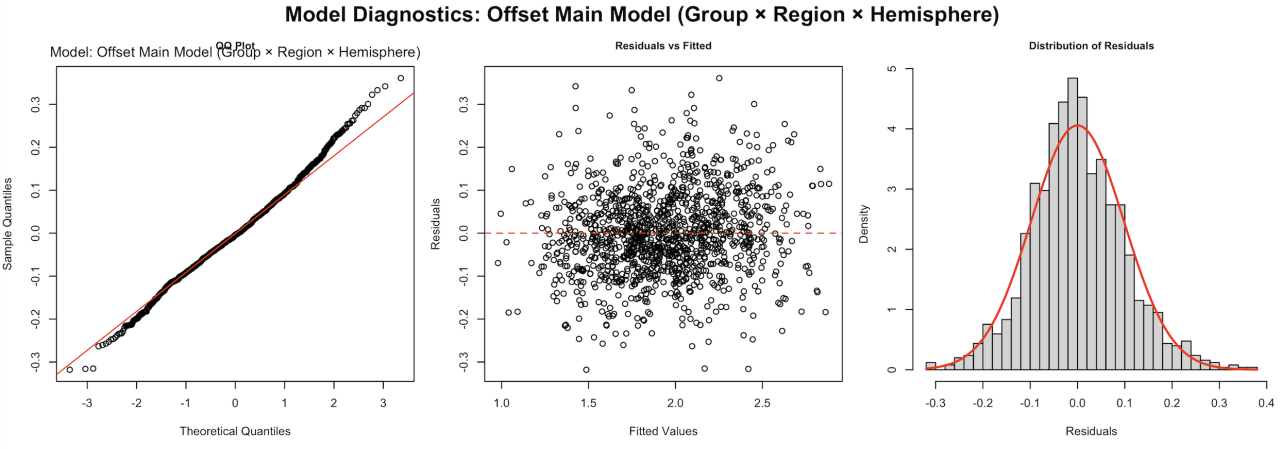


B

**Figure S3.** Model Diagnostics for Linear Mixed-Effects Models. The plots show that residuals are approximately normally distributed and homoscedastic, supporting model assumptions.
